# Microbiomic Analysis of Bacteria Associated with Rock Tripe Lichens from Alpine Areas in Eastern Alps and Equatorial Africa

**DOI:** 10.1007/s00284-024-03626-8

**Published:** 2024-03-14

**Authors:** Zichen He, Takeshi Naganuma, Ryosuke Nakai, Jun Uetake, Martin W. Hahn

**Affiliations:** 1 Graduate School of Integrated Science for Life, Hiroshima University, Higashi-Hiroshima, 739-8528 Japan; 2 Bioproduction Research Institute, National Institute of Advanced Industrial Science and Technology, Sapporo, 062-8517 Japan; 3 Field Science Center for Northern Biosphere, Hokkaido University, Sapporo, 060-0811 Japan; 4 Research Department for Limnology, Universität Innsbruck, 5310 Mondsee, Austria

## Abstract

The diversity of bacteria associated with alpine lichens was profiled. Lichen samples belonging to the *Umbilicariaceae* family, commonly known as rock tripe lichens, were gathered from two distinct alpine fellfields: one situated on Mt. Brennkogel located in the Eastern European Alps (Austria), and the other on Mt. Stanley located in the Rwenzori mountains of equatorial Africa (Uganda). The primary aim of this research was to undertake a comparative investigation into the bacterial compositions, and diversities, identifying potential indicators and exploring their potential metabolisms, of these lichen samples. Bulk genomic DNA was extracted from the lichen samples, which was used to amplify the 18S rRNA gene by Sanger sequencing and the V3-V4 region of the 16S rRNA gene by Illumina Miseq sequencing. Examination of the fungal partner was carried out through the analysis of 18S rRNA gene sequences, belonging to the genus *Umbilicaria* (*Ascomycota*), and the algal partner affiliated with the lineage *Trebouxia* (*Chlorophyta*), constituted the symbiotic components. Analyzing the MiSeq datasets by using bioinformatics methods, operational taxonomic units (OTUs) were established based on a predetermined similarity threshold for the V3-V4 sequences, which were assigned to a total of 26 bacterial phyla that were found in both areas. Eight of the 26 phyla, i.e. *Acidobacteriota*, *Actinomycota*, *Armatimonadota*, *Bacteroidota*, *Chloroflexota*, *Deinococcota*, *Planctomycetota*, and *Pseudomonadota*, were consistently present in all samples, each accounting for more than 1% of the total read count. Distinct differences in bacterial composition emerged between lichen samples from Austria and Uganda, with the OTU frequency-based regional indicator phyla, *Pseudomonadota* and *Armatimonadota*, respectively. Despite the considerable geographic separation of approximately 5430 km between the two regions, the prediction of potential metabolic pathways based on OTU analysis revealed similar relative abundances. This similarity is possibly influenced by comparable alpine climatic conditions prevailing in both areas.

**Supplementary Information:**

The online version contains supplementary material available at 10.1007/s00284-024-03626-8.

## Introduction

Rock tripes are the rock-dwelling lichen species belonging to the genus *Umbilicaria* Hoffm., 1789 (*Ascomycota*, *Lecanoromycetes*, *Umbilicariales*, *Umbilicariaceae*) [1]. The genus *Umbilicaria*, consisting of eight subgenera [2], comprises > 70 species according to the NCBI Taxonomy Brower [3], with new species and new records of occurrences reported in recent years [4–8]. The *Umbilicaria* lichens mainly inhabit mountains and fellfields worldwide [9], including Antarctic ice-free areas [10–12]. *Umbilicaria* populations including *Umbilicaria antarctica*, *U*. *kappeni*, *U*. *decussata,* and *U*. *umbilicarioides* have colonized and re-lichenized in the Antarctic Peninsula multiple times independently [13]. Microbiomes of maritime and east Antarctic *Umbilicaria* exhibit bioclimatic variation [14]. Similarly, *U. pustulata* (syn. *Lasallia pustulata*) in Mediterranean Sardinia shows bioclimatic adaptation along an elevational cline between 176 and 1303 m above sea level (a.s.l.) [15], with its holo-genome, i.e. an entire genome of myco-/photobionts, and microbiome investigated [16]. Bacteria associated with lichens including *Umbilicaria* have been studied by culture-based and culture-independent methods [17]. Bacteria in three samples of *Umbilicaria cylindrica* at about 1800 m a.s.l. of Mt. Handalm, Austria, were investigated microscopy-based by using fluorescence in situ hybridization (FISH) by using taxon-specific probes [18]. However, microbiomes of *Umbilicaria* inhabiting alpine areas of > 2000 m a.s.l. have rarely been profiled. Last year, an article reported the microbiomes of a total of 12 samples of *U. pustulata* and *U*. *hispanica* between 700 and 2100 m a.s.l. on the southern incline of the Sierra de Gredos, a mountain range located in the Sistema Central region of Spain [19]. By contrast, the sampling points at the Eastern Alps in this study were all higher than 2500 m a.s.l. As regards sampling points of Equatorial Africa, they were all higher than 4700 m a.s.l. Previously, a study analyzed the microbiomes of five samples of the beard lichen *Usnea* between 2989 and 4048 m on Nyingchi in Tibet Autonomous Region (Southwest China) [20]. Here, we report microbiomes of alpine *Umbilicaria* from Mt. Stanley, Uganda, and Mt. Brennkogel, Austria, to examine whether similar Alpine bioclimates may affect similarities in taxonomic compositions or predicted metabolic features of lichen-associated bacteria. This study reports the part of bacterial 16S rRNA gene, i.e. bacterial V3-V4 sequences to profile the microbiomic compositions and to predict metabolic functions of bacteria associated with a total of 16 samples of alpine *Umbilicaria* lichens.

## Materials and Methods

### Collection of Rock Tripe Lichen Samples

**Fig. 1:**
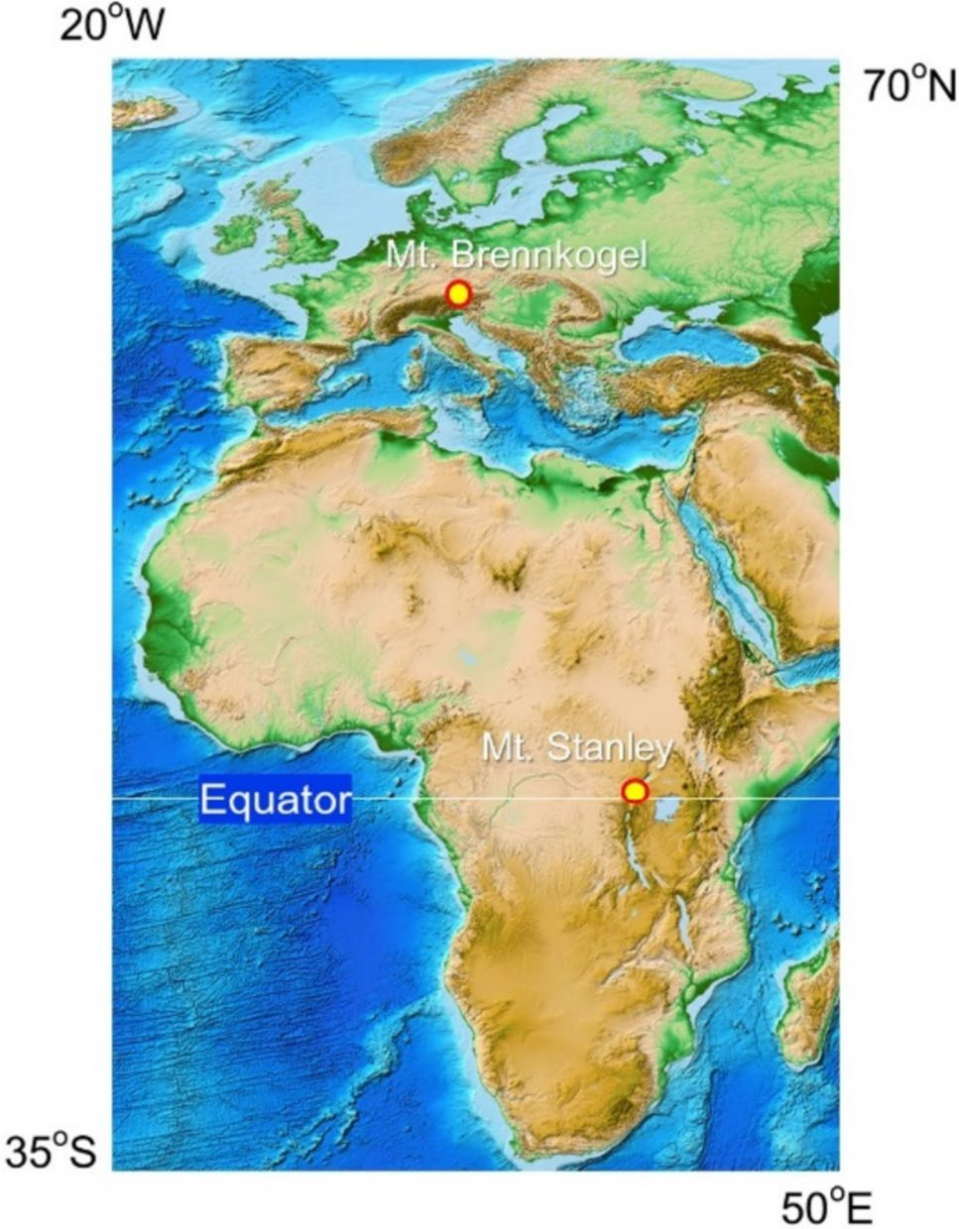
Geographical distribution of rock tripe lichen sampling sites on Mt. Brennkogel, Eastern Alps (Austria) and Mt. Stanley, Rwenzori Mountains, equatorial Africa (Uganda). The calculated distance between the two areas is calculated as about 5430 km. The map image is adapted from the digital composite model named ETOPO1 Global Relief Model [22]

The target lichen samples belonging to the genus *Umbilicaria* primarily found on fellfield rocks, were gathered from two geographically separated alpine biogeographic areas: five samples from Mt. Brennkogel (sampled at elevations of about 2600 m) of the Glockner Group located within the Nationalpark Hohe Tauern in the Eastern Alps (Austria) on 30 July 2019; and 11 samples from Mt. Stanley (sampled at elevations of about 4700 m) located within the Rwenzori Mountains National Park in equatorial Africa (Uganda) on 18 February 2014 (Fig. 1). Photographs of the thalli taken at the collection sites are shown in Figure S1. Detailed geographical data, encompassing coordinates (longitude and latitude), as well as the altitudes of the respective sampling sites, have been documented in Table 1 for reference. The calculated distance between these two distinct regions amounts to approximately 5430 km, as computed utilizing the Great Circle Calculator [21].

**Table 1:** Sampling sites where rock tripe lichens are found in fellfield environments

Region	Area	Latitude	Longitude	Altitude (m)	Sample Code
Eastern Alps (Austria)	Mt. Brennkogel Glockner Group	47° 05′ 03.2″ N	12° 50′ 15.2″ E	2622	A01, A02, A03
		47° 04′ 57.7″ N	12° 50′ 28.7″ E	2594	A04, A05
Equatorial Africa (Uganda)	Mt. Stanley Rwenzori Mountains	00° 22′ 31″ N	29° 52′ 40″ E	4750	U01, U02, U03, U04
		00° 22′ 34″ N	29° 52′ 38″ E	4735	U05, U06, U07, U08
		00° 22′ 28″ N	29° 52′ 34″ E	4705	U09, U10, U11

Geographic distribution of rock tripe lichens residing in fellfields, the sampling sites along a trekking route of Mt. Brennkogel in Eastern Alps, Austria, and the fellfields along the termini of near-summit glaciers of Mt. Stanley of the Rwenzori Mountains, Uganda. GPSMAP62S (Garmin, Olathe, Kansas, USA) was used to calculate geographic coordinates and altitudes. The sites in Mt. Stanley are close to St. 1 and St. 2 of a previous study [23]

Rhizines of the target lichen samples were meticulously removed using a surgical blade that had been sterilized with a flame, ensuring aseptic conditions, from the fellfield rocks. Then, the intact lichen thalli were promptly placed into Nasco (Atkinson, USA) products Whirl–Pak packages which had been sterilized previously, using tweezers that were also flame-sterilized. Following collection, the lichen thalli were dried by exposure to air on the sampling position and maintained in darkness during both shipment and storage phases, and then they were frozen and stored around a temperature range of − 25 °C to − 30 °C within a laboratory freezer, awaiting the subsequent extraction of bulk genomic DNA. The amounts of collected thalli were not identical at each sampling site. and conditions during transportation were different. The Ugandan samples experienced tropical rainforest temperature and humidity during descending and transport to the airport for 5 days. The Austrian samples were kept in cool, dry, and dark places (unfrozen to avoid thawing during transportation). All samples were frozen upon arrival in the lab in Japan.

### Bulk Genomic DNA Extraction from Lichen Thalli

Around one gram of collected target lichen samples from each specified sampling position mentioned above was weighed and rinsed by using an autoclaved Merck Millipore system (Burlington, MA, USA) produced high-purity water, Milli-Q ultrapure. After being cut into small fragments with sterilized scissors, the rinsed target lichen samples were subsequently pulverized using an autoclaved mortar and pestle. For DNA isolation, the Nippon Gene (Tokyo, Japan) produced ISOIL Large for Beads ver.2 device was employed for the isolation of bulk genomic DNA from the finely ground collected target lichen samples by using the bead-beating method, then add facilitator of precipitation, Ethachinmate (also produced by Nippon Gene) with 70% ethanol into the bulk DNA solution, following the past outlined procedure [12]. The resulting DNA precipitate was carefully redissolved in autoclaved Milli-Q ultrapure water. Purity and quantity assessments were performed by using the Thermo Fisher Scientific (Waltham, MA, USA) produced NanoDrop 2000c. Before the PCR amplification, the DNA samples were then securely stored at a temperature of − 20 °C.

### Amplification and Sequencing of Fungal/Algal 18S rRNA Gene

Extracted genomic DNAs from lichen samples were employed to amplify the sequences of fungal/algal 18S rRNA gene (near-full-length) by using TaKaRa Bio (Kusatsu, Japan) produced polymerase chain reaction (PCR) kit, TaKaRa Ex Taq (Mg^2+^ plus Buffer). The PCR machines used for this study included three TaKaRa Bio machines, a TaKaRa PCR Thermal Cycler Dice Touch TP350, and two TaKaRa PCR Thermal Cycler PERSONAL. The primer sequences and the targeted sequences are listed in Table 2.

**Table 2:** List of primers with target sequences in this study. F and R mean forward and reverse primer

Target Sequence	Primer Designation	F/R	Length (-mer)	5′ → 3′	Expected Product Size	Ref
Fungal 18S rRNA gene	NS17UCB	F	19	CATGTCTAAGTTTAAGCAA	2.0 kbp	[24]
	NS24UCB	R	20	AAACCTTGTTACGACTTTTA		
Eukaryotic internal transcribed spacer (ITS)	ITS1F	F	22	CTTGGTCATTTAGAGGAAGTAA	600 bp	[25]
	ITS4R	R	20	TCCTCCGCTTATTGATATGC		[26]
Algal 18S rRNA gene	Euk F	F	21	AACCTGGTTGATCCTGCCAGT	1.8 kbp	[27]
	Al1700r*	R	18	CTCCTTCCTCTAGGTGGG		[28]
V3-V4 region of 16S rRNA gene	341F	F	17	CCTACGGGNGGCWGCAG	460 bp	[29]
	805R	R	21	GACTACHVGGGTATCTAATCC		[29]

^*^ Reverse-complement of Al1700f

The extracted bulk DNA was used in PCR after dilution in × 50, × 100, × 200, × 500, × 1000. For the process of fungal 18S rRNA genes amplification, the PCR protocol comprised an initial denaturation phase, 30 cycles involving denaturation/annealing/extension, and a final extension step at 95 °C for 5 min, 95 °C for 45 s/61 °C for 45 s/72 °C for 80 s, and 72 °C for 12 min, respectively. Similarly, the PCR protocol utilized for the process of algal 18S rRNA genes amplification encompassed similar initial denaturation, 30 cycles involving denaturation/annealing/extension, and a final extension step at 95 °C for 5 min, 95 °C for 45 s/53 °C for 45 s/72 °C for 80 s, and 72 °C for 12 min, respectively. In the case of internal transcribed spacer (ITS) regions of fungal sequences, the PCR protocol was structured as follows: an initial denaturation step, 30 cycles involving denaturation/annealing/extension, and a final extension step at 95 °C for 5 min, 95 °C for 30 s/54 °C for 30 s/72 °C for 50 s, and72°C for 6 min, respectively. The products with the best amplification effect will be diluted in × 50, × 100, × 200, × 500, × 1000 again for the second PCR with the same protocols. This method is based on our previous experience, and it has a good effect on *Umbilicaria* thalli washed with ultrapure water [14].

The second PCR amplification products of fungal/algal 18S rRNA gene as well as ITS region were purified using Roche (Basel, Switzerland) produced High Pure PCR Product Purification Kit. Purified products were prepared with Thermo Fisher Scientific produced BigDye Terminator v3.1 Cycle Sequencing Kit and loaded to a Thermo Fisher Scientific produced ABI 3730XL automatic DNA Sequencer for Sanger sequencing with the same primers as used for the PCR amplifications but highly purified by HPLC in Hiroshima University institution, the Department of Gene Science, belonging to Natural Science Center for Basic Research and Development (N-BARD) [12].

### Amplification and Sequencing of V3-V4 Region of Bacterial 16S rRNA Genes

The V3-V4 region of the 16S rRNA gene was amplified using purified bulk genomic DNA extracted from the lichen samples by Kapa Biosystems, Inc. (Wilmington, DE, USA) produced Kapa HiFi HotStart ReadyMix PCR kit. These amplifications were conducted with specific primers 341F and 805R (Table 2). The PCR protocol for the V3-V4 region was initiated by an initial denaturation phase, 30 cycles involving denaturation/annealing/extension, and a final extension step at 95 °C for 3 min, 95 °C for 30 s/55 °C for 30 s/72 °C for 30 s, and72°C for 5 min. Following successful PCR amplification, the resulting obtained products were subjected to purification and subsequent quality analysis. Amplicons of high quality were selected for paired-end 300 bp sequencing, facilitated by the Illumina (San Diego, CA, USA) produced Nextera XT Index Kit, conducted on the Illumina MiSeq platform. The process of sequencing took place at the molecular diagnostic companies, Environmental Research and Solutions Co. Ltd. (Kyoto, Japan), and SolGent Co. Ltd. (Daejeon, Korea).

### Sequence Data Analysis and OTU Determination

Sequences obtained from the Sanger method for 18S rRNA genes of fungal and algal partners, as well as fungal ITS regions, underwent a rigorous examination process by BioEdit biological sequence alignment editor with the ClustalW program to remove low-quality results [30, 31]. High-quality sequences were retained and assembled manually and then scrutinized by tree topology analysis to check for potential chimeras [32]. The resultant sequences, deemed reliable, were subsequently harnessed for identifying the fungal and algal partners of the lichen specimens under investigation. This identification was facilitated through BLAST search (provided by the National Center for Biotechnology Information). The BLAST search on the NCBI website uses the default parameters. The resultant max scores were > 3000, total scores were > 3000, query coverages were > 95%, and E-values were 0.0. Among the BLAST results, the sequences of fungal 18S rRNA and ITS regions were used to classify the species affiliation of lichen samples, and the sequences of algal 18S rRNA were used to identify eukaryotes that the main photosynthetic partners in the lichen samples.

The reads of the V3-V4 region generated through the MiSeq method were contained in fastq files which were uploaded to the EzBioCloud-provided Microbiome Taxonomic Profiling (MTP) pipeline to be processed and analyzed (https://www.ezbiocloud.net/contents/16smtp; accessed on 14 July 2023) [33]. Concisely, the paired-end reads were merged using the overlapping sequence information and trimmed to remove the remaining primer sequences by using EzBioCloud in-house algorithms and scripts. Any unmerged reads, reads with lengths of < 100 bp or > 2,000 bp, and ambiguous reads with low average quality scores (less than 25) were detected and discarded. For the reads that successfully passed through the quality filtering, identical sequences were deduplicated to reduce computational time. These deduplicated sequences were then subjected to taxonomic assignment, utilizing the EzBioCloud prokaryotic sequence database of 16S rRNA gene PKSSU4.0. In this process, the target taxon options were restricted to "Bacteria." Any uncultured taxonomic category in the PKSSU4.0 database was provisionally assigned a hierarchical compound name based on DDBJ/ENA/GenBank/PacBio sequence accession number, using suffixes such as "_s", "_g", "_f", "_o", "_c" and "_p" for species, genus, family, order, class, and phylum, respectively. Clear taxonomic rank boundaries were set based on 16S rRNA gene sequence identity cut-off values as follows: 97%, 94.5%, 86.5%, 82.0%, 78.5%, and 75.0% for species, genus, family, order, class, and phylum, respectively [34]. Unclassified reads of species or higher rank below these cut-off values were tentatively appended with the suffix "uc". Any read that failed to match any reference sequence by the database searching with a 97% similarity cut-off value was submitted to chimera sequence detection algorithms (including reference-dependent detection and de novo detection). This process was conducted utilizing the reference database (https://help.ezbiocloud.net/mtp-pipeline/; accessed on 14 July 2023) available from EzBioCloud. Any read identified as a chimera through this analysis was subsequently removed. Finally, the remaining V3-V4 reads were picked and clustered into OTUs at the cut-off value of 97% identity [33], which has been found to offer improved universality when compared to the 98% [35] proposals. In the OTU picking process, singleton reads and eukaryotic plastid reads were ignored. Species level is a possibility extended from a genus-level analysis. However, it should be noted that the analysis results at the species level using V3-V4 were for reference only and they were not regarded as completely accurate.

### Diversity Indices and Bioinformatic Analyses of OTUs

To assess the richness and evenness of bacterial operational taxonomic units (OTUs) in the lichen samples, rarefaction curve analysis and computation of alpha-diversity indices encompassing Chao1 richness, Shannon indices, and Simpson indices were carried out. These analyses were conducted utilizing the EzBioCloud MTP pipeline. Notably, it's important to highlight that the Chao1 index incorporated singleton OTUs in its calculations.

The assessment of beta-diversity within alpine operational taxonomic units (OTUs) encompassed the visualization of results through principal component analysis (PCA) and constructed a hierarchical clustering dendrogram based on the UniFrac distance measure [36]. Indicator OTUs, which distinguish microbiomes through statistically significant frequency differences in two distinct Alpine regions, were identified using the linear discriminant analysis (LDA) and the LDA Effect Size (LEfSe) algorithm, which was based on relative read frequencies. (http://huttenhower.sph.harvard.edu/galaxy/; accessed on 14 July 2023) [37, 38]. While previous investigations of lichen-associated bacteria commonly established a default threshold LDA score of 2 [39, 40], this study adopted threshold scores of 3 and 4 to concentrate the analysis on significant differences observed in substantial statistical indicators between the two distinct sampling regions. Indicators exhibiting an LDA score exceeding 4 underwent additional scrutiny to assess differential abundances between the two regions, which was carried out using the Analysis of Compositions of Microbiomes with Bias Correction (ANCOM-BC) methodology [41].

The exploration of potential metabolic pathways using OTUs from lichen-associated bacteria, originating from the two separate sampling regions was conducted, which involved the prediction of known metabolic pathways by the metabolic pathways catalog available in the Kyoto Encyclopedia of Genes and Genomes (KEGG; http://www.genome.jp/kegg/; accessed on 14 July 2023) [42]. The database structure is provided by an extendable network visualization and analysis tool Visualisation and Analysis of Networks containing Experimental Data (VANTED; https://www.cls.uni-konstanz.de/software/vanted/; accessed on 14 July 2023) [43]. The V3-V4 sequence-based KEGG-search was performed by the Phylogenetic Investigation of Communities by Reconstruction of Unobserved States 2.0 (PICRUSt 2.0; https://huttenhower.sph.harvard.edu/picrust/; accessed on 14 July 2023) [44]. A visual bioinformatics analysis tool for PICRUSt 2.0 was provided by OmicStudio online (https://www.omicstudio.cn/tool; accessed on 14 July 2023). Any non-prokaryotic metabolic pathways included in the analysis results of potential metabolisms were discarded manually.

The sequences derived from the Sanger method for 18S rRNA genes of fungal and algal partners, as well as fungal ITS regions, have been duly archived in DDBJ/ENA/GenBank database, and assigned accession numbers are as LC730211–LC730226 for sequences of fungal 18S rRNA gene, LC730227–LC730242 for sequences of algal 18S rRNA gene, as well as LC744762–LC744777 for fungal ITS regions sequences. The dataset containing V3-V4 regions generated through the MiSeq method in this study has been deposited in the DDBJ Sequence Read Archive (DRA) and the assigned accession numbers are DRA014883 and DRA014939 respectively. Associated BioSample numbers are SAMD00535656–SAMD00535666 and SAMD00547134–SAMD00547138, respectively, under the same BioProject number PRJDB14357. The samples and accession numbers are correspondingly recorded in Tables S1–S3, as well as S7. Detailed results of MiSeq read counts and taxonomic classification (at the genus level) recovered from the studied lichen samples are shown in Table S4.

## Results

### Identification of Rock Tripe Lichen-forming Fungi and Algae

The sequences of fungal/algal 18S rRNA gene (near-full-length) from the sampled lichens in Austria and Uganda were BLAST-searched. The analysis of fungal partners across all 16 samples indicated that they all belonged to the genus *Umbilicaria* (fall under the *Ascomycota* phylum), and matching rates of 99.30% or greater were observed, along with query coverages of 97% or higher (Table S2). The sequence similarity between the Austrian and Ugandan mycobionts ranged from 99.18% to 99.88%. The sequences of fungal 18S rRNA gene (near-full-length), only ITS regions, and ITS regions with elongation from the sampled lichens in Austria and Uganda were BLAST-searched (Table S2–S4). The analysis of fungal partners across all 16 samples indicated the closest relationship to the *Umbilicaria rhizinata* voucher agrED295 or *U. aprina* voucher agrED360, with matching rates of 99.30% or greater (Table S2), the sequence similarity between the Austrian and Ugandan 18S rRNA gene ranged from 99.18% to 99.88%; *U. africana* voucher acpED473, *U. aprina* isolate AFTOL-ID 7153 and *U. aprina* isolate AFTOL-ID 7116 with all the same matching rates of 99.79% (Table S3); *U. africana* voucher acpED473, *U. aprina* isolate AFTOL-ID 7153 and *U. aprina* isolate AFTOL-ID 7116 with matching rates of 98.70% or greater (Table S4); determined by the BLAST-searched results of sequences of fungal 18S rRNA gene (near-full-length), only ITS regions, and ITS regions with elongation, respectively. It should be noted that although in the BLAST results, the highest similarities of all sequences are divided into three different species: *U*. *rhizinata*, *U*. *aprina,* and *U*. *africana*. However, *U*. *rhizinata* is a synonym of *U*. *aprina*, and *U*. *africana* was also classified in the *U*. *aprina* group [2, 7]. Therefore, according to the current classification, all of them can be collectively classified as the *U*. *aprina* group. The more detailed classification requires further research.

The examination of algal partners within the 16 samples revealed their closest affiliation to the green algal lineage *Trebouxia jamesii* (UBT-86.132E2), a widely observed photobiont in lichens [45], and matching rates of 98.46% or greater were observed in the Uganda region (Table S7). But in the Austrian samples only similarity values between 96.85% and 97.67%. The sequence similarity between the Austrian and Ugandan photobionts ranged from 96.09% to 98.26%.

### Evaluation of MiSeq-generated V3-V4 Sequences and OTUs

By utilizing Illumina MiSeq sequencing, a cumulative sum of 755,198 raw reads was initially generated from a total of 16 collected target lichen specimens. After undergoing filtering procedures, 572,363 valid paired reads remained, and these were subsequently organized into OTUs. Drawing from the analysis data present in the EzBioCloud database [33], the average length of all valid reads stood at 403.0 base pairs (bp). The sequences from the Austrian samples exhibited an average length of 404.2 bp, while those from the Ugandan samples displayed a slightly shorter mean length of 402.4 bp. Table 3 provides comprehensive details encompassing raw reads, valid reads, the counts of grouped OTUs, species, genera, families, orders, classes, and phyla derived from OTUs, as well as the mean sequence length for each sample.

**Table 3:** Each composition of lichen samples is characterized by the counts of MiSeq-generated V3-V4 region reads, 97% similarity-based OTUs, OTU-derived species, genera, families, orders, classes, and phyla

Sample	Raw	Valid	OTU	Species	Genus	Family	Order	Class	Phylum	Mean length (bp)
	**read**	**read**								
A01	44,775	22,950	339	188	102	57	33	21	12	404.8
A02	35,603	21,487	309	144	73	42	28	18	8	403.7
A03	22,890	16,157	419	272	125	66	43	27	12	403.8
A04	36,988	22,892	443	241	122	68	44	27	12	404.0
A05	43,148	13,105	650	504	249	113	60	35	13	404.8
Sub-total	183,404	96,591	1,399	794	347	143	76	44	17	404.2
U1	39,782	36,029	410	278	144	69	45	32	16	402.8
U2	34,625	33,078	462	342	186	83	55	36	14	402.7
U3	48,621	47,012	354	248	134	58	40	27	11	401.7
U4	65,657	64,214	691	465	231	101	63	37	15	401.4
U5	58,739	49,238	657	468	226	103	70	44	16	399.1
U6	60,226	59,095	451	321	164	69	47	31	14	401.8
U7	83,129	59,812	555	425	237	115	70	44	20	404.0
U8	60,188	45,252	164	137	73	45	32	25	11	401.6
U9	49,882	17,626	133	108	64	46	33	24	13	403.1
U10	41,779	39,092	566	377	201	100	62	40	18	405.2
U11	29,166	25,324	206	168	84	53	35	25	11	403.3
Sub-total	571,794	475,772	1,544	1,011	458	193	109	64	25	402.4
Total	755,198	572,363	2,492	1,460	602	233	123	68	26	403.0

It is important to note that the sub-total and total counts of taxa might be reduced due to potential overlaps among samples. The dataset provides insight into the mean lengths of valid reads for each sample. The samples denoted as A01 to A05 were obtained in Austria, whereas samples labeled U1 to U11 were obtained from Uganda

The coverage observed in the rarefaction analysis signifies the ratio between the actual number of observed OTUs and the estimated OTU count. This equivalence aligns with the alpha-diversity index, Chao1. The coverages in Alpine lichen samples can be calculated from Table 5 and mean/minimum/maximum ratios were 91.75%/85.26% (in U9)/97.48% (in A02), respectively. The coverage ratios suggested valid reads generated in this study are deemed adequate for subsequent statistical and bioinformatic analyses.

Table 4 displays the distribution of taxa counts across different regions (including OTUs, species, genera, families, orders, classes, and phyla) that were exclusively identified in Austrian lichen samples, those found solely in Ugandan samples, and those observed in samples from both regions. Observed OTUs, species, and genus ranks showed higher percentages of region-specific features, while the order, class, and phylum ranks were shown in both regions with more than half of region-common features. The findings revealed a similarity between the two regions at higher taxonomic ranks, accompanied by distinctiveness unique to each region at lower taxonomic levels.

**Table 4:** The counts of assigned OTUs, as well as OTU-derived species, genera, families, orders, classes, and phyla, are detailed for the categories of solely Austrian, and solely Ugandan, and shared across both regions

Distribution	Observed OTU	Species	Genus	Family	Order	Class	Phylum
Only in Austria	948	449	144	40	14	4	1
Only in the Uganda region	1093	666	255	90	47	24	9
Common to both regions	451	345	203	103	62	40	16
Total	2492	1460	602	233	123	68	26

These cumulative numbers align with the totals presented in Table 3

### Taxonomic Composition of Lichen-associated Bacterial Communities

**Fig. 2:**
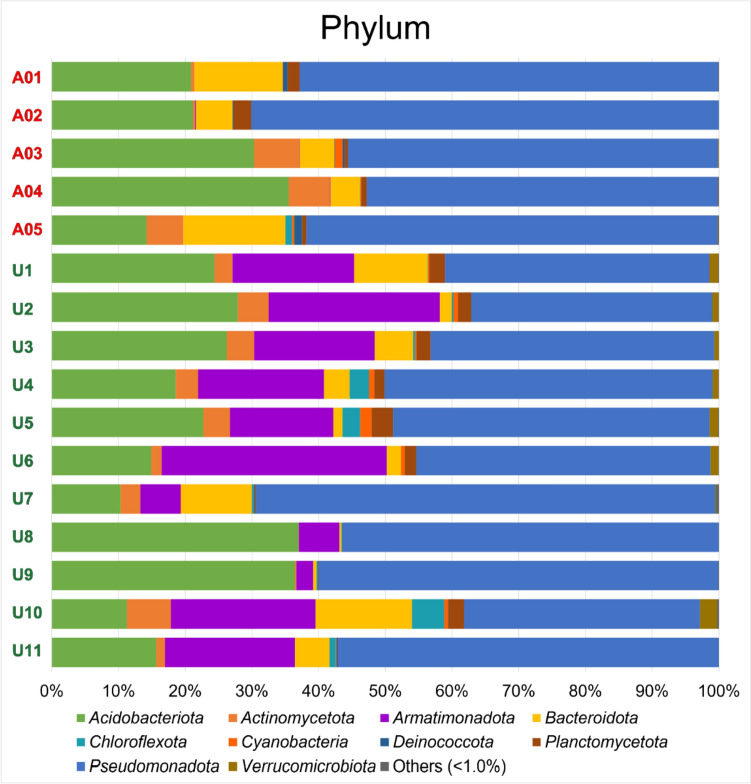
The bacterial compositions on phylum rank of OTUs, derived from collected target lichen samples collected in Austria (A01 to A05) and Uganda (U1 to U11), are depicted. A total of eight different phyla were identified with read abundances exceeding 1% of the total read count for each region. Compositions of bacterial classes, orders, families, genera, and species are shown in Figures S3–S6

Figure 2 presents the compositions of bacterial phyla, analyzed from the OTUs within the collection of 16 lichen samples. In total eight phyla of bacteria were found to be common features with read frequencies of > 1% in all 16 samples. Each lichen sample contained 8–20 bacterial phyla (Table 3) including 5–15 phyla less than 1% of read frequencies. According to modern nomenclature, The popular phyla were sorted by name alphabetically as follows: *Acidobacteriota*, *Actinomycota*, *Armatimonadota*, *Bacteroidota*, *Chloroflexota*, *Deinococcota*, *Planctomycetota*, and *Pseudomonadota*.

### Alpha and Beta Diversity

To assess the OTU richness of each collected target lichen sample, alpha-diversity indices were employed, encompassing computations for Chao1 richness, Shannon indices, and Simpson indices. These computed results are presented in Table 5. Chao1 richness values and Shannon/Simpson indices were used for estimating OTU numbers for the rarefaction curve analyses and calculations of the effective number of species (*ENS*), respectively [46]. Chao1, Shannon, *ENS* values and observed OTU numbers have positive correlations with species and evenness, and Simpson index values have negative correlations with species and evenness. As a result, the Austrian samples exhibited elevated Chao1, Shannon, and *ENS* values, alongside a greater count of observed OTUs, and lower Simpson index values. These patterns collectively indicate higher species richness and evenness within the Austrian samples. However, it is noteworthy that the Ugandan sample U10 displayed the highest values across these metrics, as illustrated in Table 3.

**Table 5:** Regarding the OTUs of bacteria obtained from five collected target lichen samples sourced in Austria (designated A01 to A05) and 11 samples from Uganda (labeled U1 to U11), alpha-diversity indices encompassing Chao1 richness, Shannon indices, and Simpson indices were computed

Sample	Observed OTU	Chao1	Shannon *(ENS)*		Simpson *(ENS)*	
A01	339	355.6	3.24	*25.5*	0.08	*12.5*
A02	309	317.4	3.22	*25.0*	0.08	*12.5*
A03	419	441.3	4.25	*70.1*	0.03	*33.3*
A04	443	456.3	3.84	*46.5*	0.05	*20.0*
A05	650	755.0	3.94	*51.4*	0.06	*16.7*
**Average**	**432.0**	**465.1**	**3.70**	***43.7***	**0.06**	***19.0***
U1	410	456.1	3.71	*40.9*	0.06	*16.7*
U2	462	506.8	3.76	*42.9*	0.06	*16.7*
U3	354	381.7	3.66	*38.9*	0.06	*16.7*
U4	691	740.1	3.53	*34.1*	0.09	*11.1*
U5	657	711.7	4.02	*55.7*	0.06	*16.7*
U6	451	503.0	3.03	*20.7*	0.13	*7.7*
U7	555	603.1	2.96	*19.3*	0.14	*7.1*
U8	164	187.2	1.76	*5.8*	0.26	*3.8*
U9	133	156.4	1.76	*5.8*	0.25	*4.0*
U10	566	598.4	4.58	*97.5*	0.02	*50.0*
U11	206	233.0	2.32	*10.2*	0.15	*6.7*
**Average**	**422.6**	**461.6**	**3.19**	***33.8***	**0.12**	***14.3***

Furthermore, from Shannon and Simpson indices, effective numbers of species (*ENS*) were derived to enhance insights into the diversity metrics

Due to different calculation methods, Shannon/Simpson indices cannot be used to estimate bacterial species richness. Comparatively, Chao1 values were close to estimated OTU numbers and may better represent species richness in the case of large sample sizes, as reported in other studies [47, 48].

**Fig. 3:**
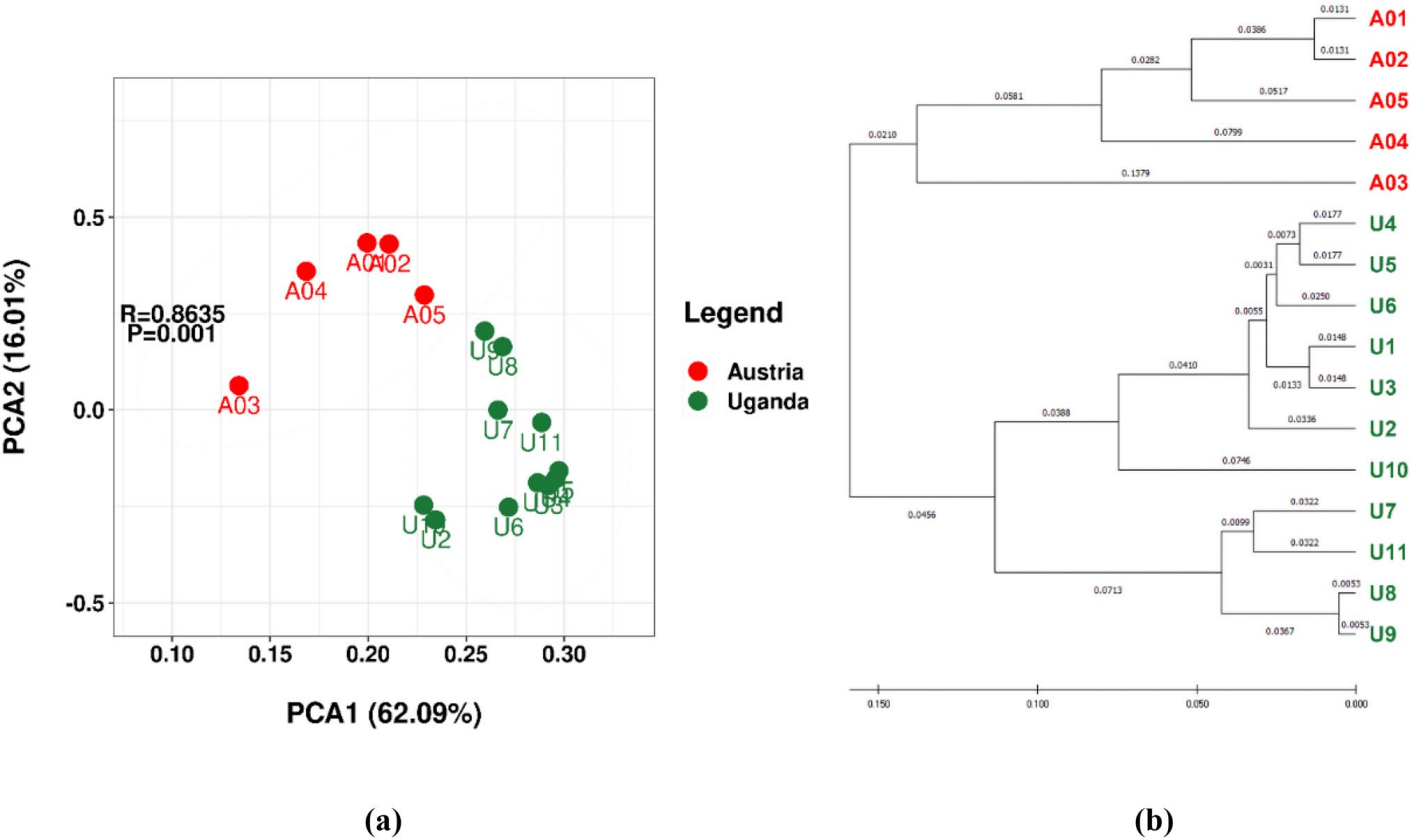
Depicting the bacterial species originating from lichen samples gathered in Austria (denoted in red) and Uganda (denoted in green), the PCA plot (**a**) and hierarchical clustering dendrogram (**b**) offer visual insights. Furthermore, a set of PCA plots is presented, each corresponding to the genus, family, order, class, and phylum ranks, and these visualizations can be found in Figure S7

Beta diversity was used for evaluating similarity/dissimilarity between different samples including PCA and hierarchical cluster analysis, which both showed clear regional separation of microbiomes obtained from Austria and Uganda at the species rank. Utilizing PCA analysis, a distinct regional demarcation was evident, particularly at the genus level. However, in Figure S7, this differentiation was not as pronounced among the various sampling locations within Austria (A01 to A05) and a subset of Uganda (U7 to U9) when examined at the family, order, class, and phylum ranks, as demonstrated (Fig. 3).

**Fig. 4:**
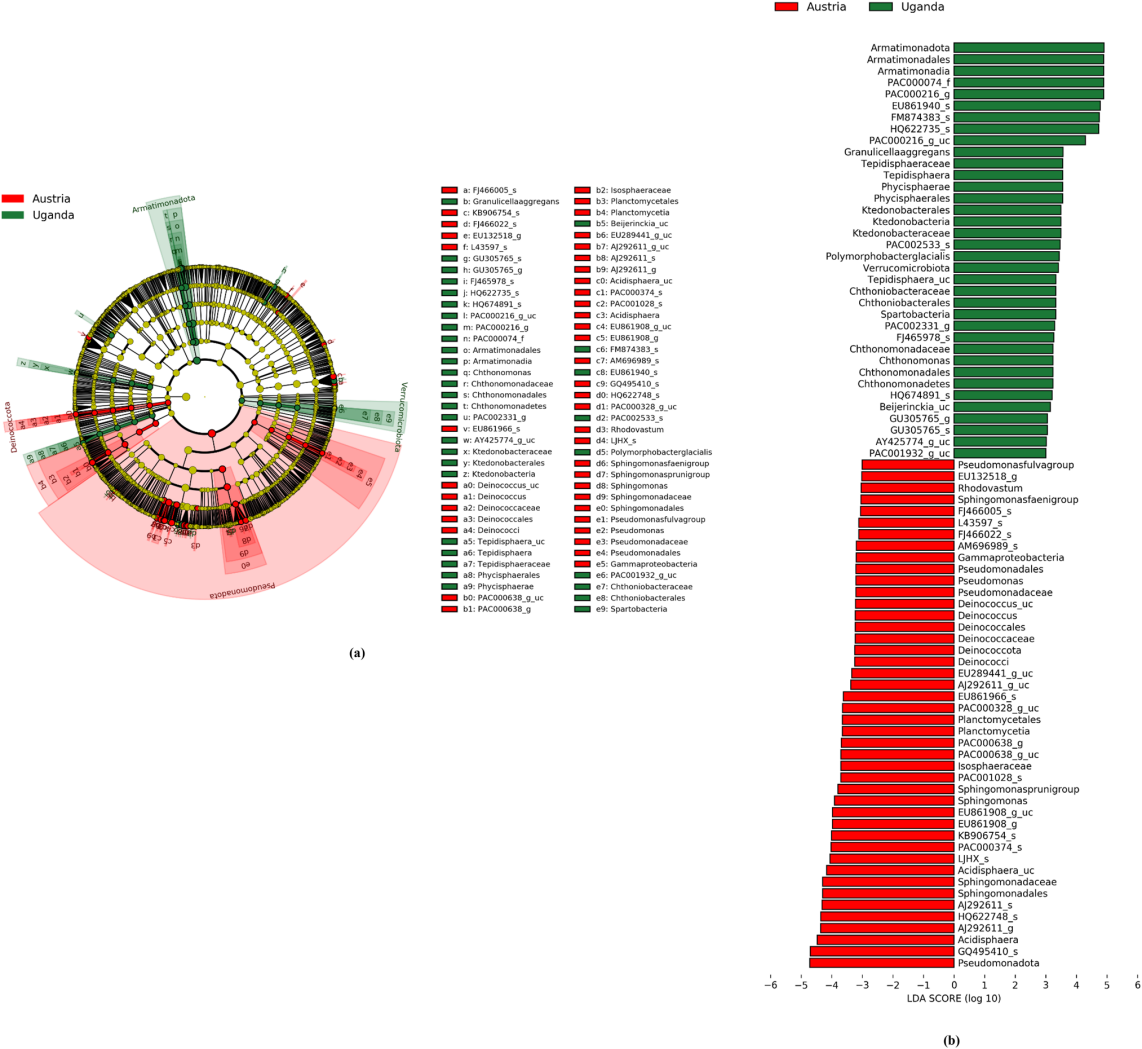
The LEfSe cladogram **(a)** and bar plot **(b)** to visualize taxonomic indicator bacteria that are specific to the regions of Austria (denoted in red) and Uganda (denoted in green) about the collected lichen samples. This cladogram features an innermost node representing the domain of Bacteria, followed by concentric nodes indicating phylum, class, order, family, genus, and species. Nodes or shaded areas in red and green signify significantly higher relative abundances of taxa. The size of each node circle is proportional to the abundance of reads attributed to the corresponding taxon

Utilizing LDA, the calculation of regional distinctiveness among OTUs was conducted and subsequently marked. This distinctiveness was further identified through LEfSe, and the resultant indicator OTUs or higher taxa were visually represented within the phylogenetic cladogram as illustrated in Fig. 4. Notably, significant indicators, categorized in Table 6, were chosen with the criterion of setting the LDA score threshold to 4. Austria exhibited significant indicators, including OTU KB906754_s (*Edaphobacter* sp.), *Acidisphaera*_uc, PAC000374_s (*Acidisphaera* sp.), AJ292611_s (an unidentified species within the *Acetobacteraceae* family), GQ495410_s (an unidentified species within the *Acetobacteraceae* family), HQ622748_s (an unidentified species within the *Acetobacteraceae* family), LJHX_s (*Polymorphobacter* sp.), as well as the *Acidisphaera* genus and AJ292611_g (an unidentified genus within the *Acetobacteraceae* family), the *Sphingomonadaceae* family, the *Sphingomoadales* order, and the *Pseudomonadota* phylum.

**Table 6:** Distinctive indicator OTUs and taxa, characterized by LDA scores exceeding 4, were pinpointed within the assigned OTU diversity of both Austria and Uganda

Region	Code in	Rank of indicator						LDA	*p-value*
	Figure 4	Phylum	Class	Order	Family	Genus	Species	Score	
Austria	c	*Acidobacteriota*	*Acidobacteriia*	*Acidobacteriales*	*Acidobacteriaceae*	*Edaphobacter*	KB906754_s	4.01	0.003
	-	*Pseudomonadota*						4.72	0.047
	c3	*Pseudomonadota*	*Alphaproteobacteria*	*Rhodospirillales*	*Acetobacteraceae*	*Acidisphaera*		4.47	0.002
	c0	*Pseudomonadota*	*Alphaproteobacteria*	*Rhodospirillales*	*Acetobacteraceae*	*Acidisphaera*	*Acidisphaera*_uc	4.16	0.006
	c1	*Pseudomonadota*	*Alphaproteobacteria*	*Rhodospirillales*	*Acetobacteraceae*	*Acidisphaera*	PAC000374_s	4.02	0.002
	b9	*Pseudomonadota*	*Alphaproteobacteria*	*Rhodospirillales*	*Acetobacteraceae*	AJ292611_g		4.36	0.002
	b8	*Pseudomonadota*	*Alphaproteobacteria*	*Rhodospirillales*	*Acetobacteraceae*	AJ292611_g	AJ292611_s	4.32	0.001
	c9	*Pseudomonadota*	*Alphaproteobacteria*	*Rhodospirillales*	*Acetobacteraceae*	PAC000328_g	GQ495410_s	4.71	0.002
	d0	*Pseudomonadota*	*Alphaproteobacteria*	*Rhodospirillales*	*Acetobacteraceae*	PAC000328_g	HQ622748_s	4.36	0.008
	e0	*Pseudomonadota*	*Alphaproteobacteria*	*Sphingomonadales*				4.29	0.027
	d9	*Pseudomonadota*	*Alphaproteobacteria*	*Sphingomonadales*	*Sphingomonadaceae*			4.29	0.027
	d4	*Pseudomonadota*	*Alphaproteobacteria*	*Sphingomonadales*	*Sphingomonadaceae*	*Polymorphobacter*	LJHX_s	4.06	0.036
Uganda	-	*Armatimonadota*						4.91	0.002
	p	*Armatimonadota*	*Armatimonadia*					4.90	0.002
	o	*Armatimonadota*	*Armatimonadia*	*Armatimonadales*				4.90	0.002
	n	*Armatimonadota*	*Armatimonadia*	*Armatimonadales*	PAC000074_f			4.89	0.002
	m	*Armatimonadota*	*Armatimonadia*	*Armatimonadales*	PAC000074_f	PAC000216_g		4.89	0.002
	j	*Armatimonadota*	*Armatimonadia*	*Armatimonadales*	PAC000074_f	PAC000216_g	HQ622735_s	4.73	0.002
	l	*Armatimonadota*	*Armatimonadia*	*Armatimonadales*	PAC000074_f	PAC000216_g	PAC000216_g_uc	4.29	0.002
	c8	*Pseudomonadota*	*Alphaproteobacteria*	*Rhodospirillales*	*Acetobacteraceae*	PAC000327_g	EU861940_s	4.78	0.002
	c6	*Pseudomonadota*	*Alphaproteobacteria*	*Rhodospirillales*	*Acetobacteraceae*	PAC000328_g	FM874383_s	4.75	0.015

Uganda exhibited significant indicators, including OTU HQ622735_s (an unidentified species within the *Armatimonadales* order), PAC000216_g_uc (an unidentified species within the *Armatimonadales* order), EU861940_s (an unidentified species within the *Acetobacteraceae* family), FM874383_s (an unidentified species within the *Acetobacteraceae* family), PAC000216_g (an unidentified genus within the *Armatimonadales* order), PAC000074_f (an unidentified family within the *Armatimonadales* order), as well as the *Armatimonadales* order, the *Armatimonadia* class, and the *Armatimonadota* phylum.

**Fig. 5:**
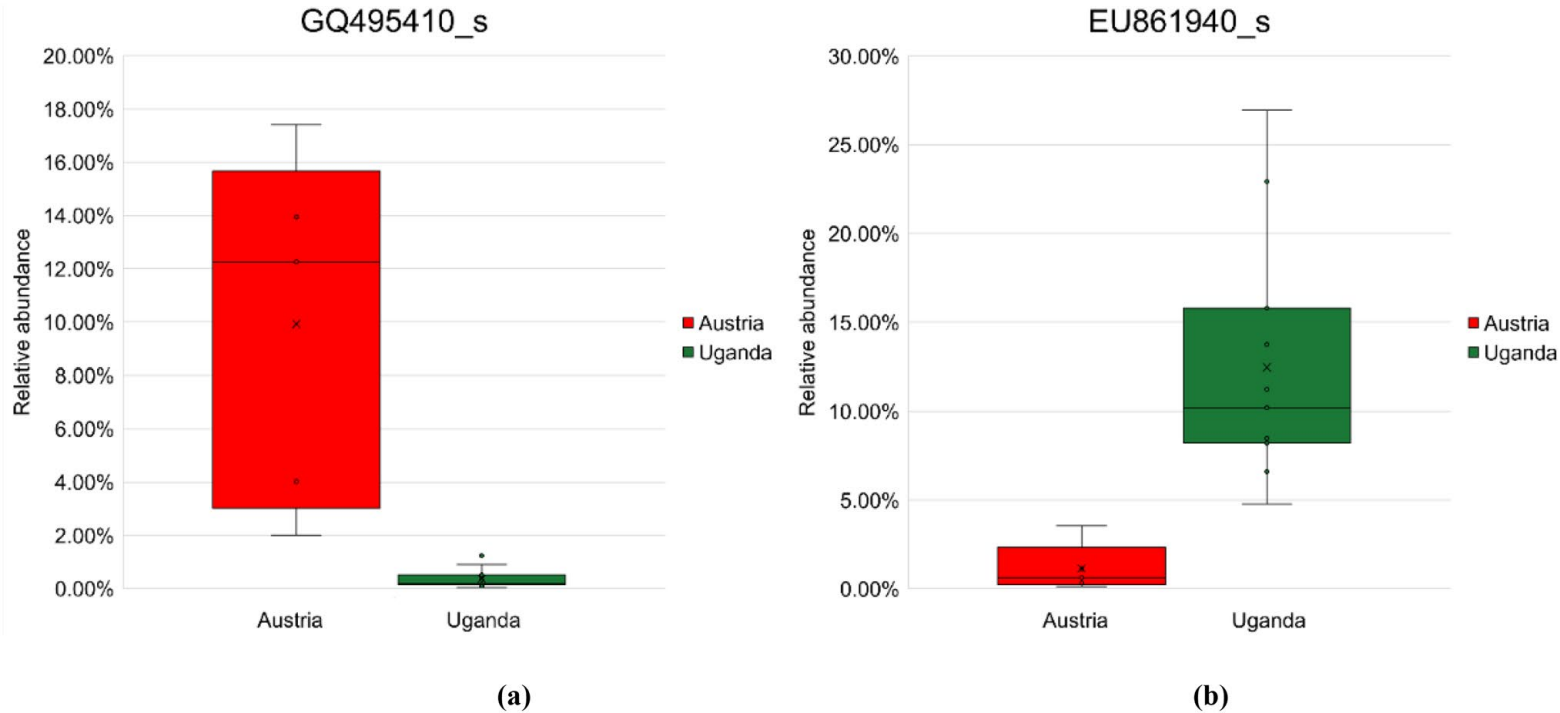
Significant dissimilarities in relative abundances of specific OTU-derived indicators (with *P* < 0.05), exhibiting the highest LDA scores, were subjected to ANCOM-BC analysis, discerning between Austria (denoted in red) and Uganda (denoted in green). (**a**), the most pronounced indicator in Austria, GQ495410_s, belonged to the genus PAC000328_g of the phylum *Pseudomonadota*. (**b**), the most pronounced indicator in Uganda, EU861940_s, belonged to the genus PAC000327_g of the phylum *Pseudomonadota*. Additional noteworthy indicator OTUs are depicted in Figure S8

On the rank of species, a total of 11 indicator OTUs (seven from Austria and four from Uganda) were identified with an LDA score > 4, which is shown in Table 6. Reducing the threshold to 3 led to the identification of a combined total of 36 indicator OTUs (14 from Austria and 22 from Uganda) at the species level, which were subsequently employed for the differential abundance analysis using ANCOM-BC. Discerned through the highest LDA scores, have been visualized in Fig. 5, while additional indicators are depicted in Figure S8.

The metabolic pathways of bacteria associated with lichens in two distinct sampling regions were predicted. Utilizing PICRUSt 2.0, the analysis involved a set of 36 indicator OTUs at the species level, distinguished by LDA scores exceeding 3. Subsequently, the representative sequences of these indicator OTUs were mapped onto KEGG metabolic maps, allowing for the visualization of essential metabolic pathways.

In Level 1 of the KEGG database classification, which represents the hugest metabolic categories within the KEGG database, each indicator OTU was projected onto five major pathways. These encompassed metabolism, genetic information processing, environmental information processing, unclassified, and cellular processes, sorted from largest to smallest, and all organized based on their respective relative abundances. Among these, the "metabolism" pathway showed the highest relative abundances as higher than 50% of OTUs of both sampling regions.

In Level 2, which encompasses sub-metabolic categories within the KEGG database, each indicator OTU was assigned to 25 distinct pathways (as shown in Figure S10). Which In Level 3, representing the finer metabolic categories within the KEGG database, each indicator OTU was associated with a total of 193 pathways (as shown in Figure S11). The most significant difference between Austria and Uganda in level 2 and level 3 metabolic pathways is only 1.0%

**Fig. 6:**
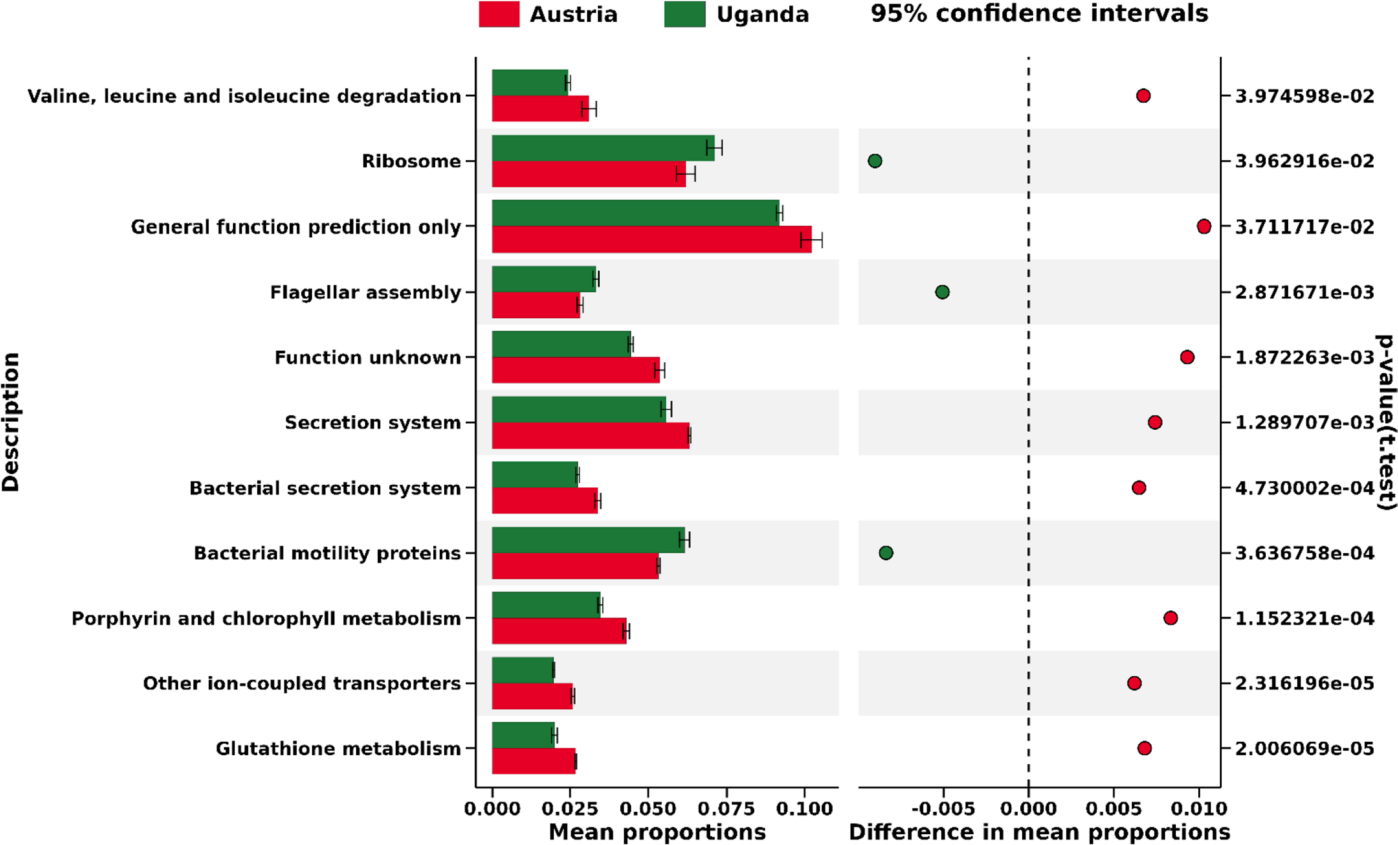
Snapshot of metabolic pathways on KEGG Level 3 identified within the indicator OTUs from Austria (denoted in red) and Uganda (denoted in green). The horizontal axis represents the average proportions of each pathway, facilitating a comparison between the two regions. Pathway selection was guided by a cut-off criterion of a mean proportion distance greater than |0.0015| between the two regions. Noteworthy distinctions, marked by significant differences at *P* < 0.05, are indicated on the right side

In addition, only 11 KEGG Level 3 metabolic pathways, which were selected by the mean proportions’ distances between two regions of >|0.0015| (significant differences at *P* < 0.05), were found among the indicator OTUs from Austria and Uganda (Fig. 6), suggesting that metabolic pathways predicted from the two regions were relatively similar.

## Discussion

Previous studies have reported the distribution of rock tripe lichens containing fungal partners (mycobionts) belonging to the *Umbilicaria* genus and algal partners (photobionts) belonging to the *Trebouxia* genus are distributed across diverse environments, even including Antarctic ice-free areas [12–14]. Given the relatively high resolution of the ITS region sequences, it was better to use in this study the sequences of the 18S rRNA gene only for the classification of the mycobionts at the genus level, i.e. classification into the genus *Umbilicaria.* While the classification at the species rank was performed by using sequences of the ITS region (Table S2–S5). Furthermore, according to importance, verifiability, and recordability, we describe those classifications as voucher specimens rather than isolate specimens although the top-hit species were different as shown in Table S5. By considering the above reasons, even though some potential variations in sequences of the 18S rRNA gene were observed in the studied *Umbilicaria* specimens during this study period, we tentatively presumed *Umbilicaria* spp. related to (> 99% ITS similarity) *U. africana* voucher acpED473 (*U. africana* relatives), as reported for other *Umbilicaria* species [14, 16]. The sequence similarity between the Austrian and Ugandan mycobionts ranged from 99.18% to 99.88%, showing that they are similar in terms of their 18S rRNA gene sequences, though additional sequence data of the ITS region is needed. Regardless of the result of fungal classification, all the fungal partners from lichen samples in this study tend to be classified as the same species according to ITS sequencing results. As mentioned in Sect. "Identification of Rock Tripe Lichen-forming Fungi and Algae", they can all be classified into the *U*. *aprina* group [2, 7]. However, the classification in the database cannot be ignored, and the more detailed classification of the lichen samples in this study requires further research. In addition, we searched and counted the number of sequences annotated as *U. aprina*, *U. rhizinata,* or *U. africana* sequences in the NCBI database (Table S6). Compared with *U. aprina*, only very few sequences assigned to the species *U. rhizinata* and *U. africana* were deposited, especially ITS region sequences of these two taxa are very rare. We believe that the influence of the database cannot be ignored, however, a potential problem of misclassified sequences present in the database cannot be solved by this study.

At both the species and genus levels, the bacterial OTUs associated with mycobionts related to *U. africana* exhibited distinct differences between the Austrian and Ugandan samples. Both PCA and hierarchical clustering analysis results illustrated this disparity (as shown in Fig. 3 and Figure S7). Interestingly, even among the microbiomes of the identical lichen species (*U. africana* relatives), a dispersed rather than clustered pattern was observed on the dendrogram, distinguishing the different sampling regions of Mt. Brennkogel in the Eastern Alps and Mt. Stanley in the Rwenzori Mountains. In other words, regardless of the same *Umbilicaria* species, the bacterial OTUs displayed distinct clustering based on geographical regions at least at the genus rank.

The algal 18S rRNA gene sequences from Austria and Uganda showed 96.85% − 97.67% and 98.46% − 99.46% similarities to *Trebouxia jamesii* (UBT-86.132E2), respectively. The sequence similarity of the Austrian and Ugandan-derived sequences to each other in the range of 96.09% to 98.26% may even suggest that the algae present in the Austrian and Ugandan samples belong to different species within the genus *Trebouxia*. Combining PCA and hierarchical clustering analysis results, it is speculated that different species of algae can determine the different compositions of the bacterial biomes.

The total valid reads from Austrian samples were only about 14% of the overall total raw reads, contrasting with 71% from Ugandan samples. The average (per sample) read number in the five Austrian samples was 19,116, lower than that of the 11 Ugandan samples, 43,252. The difference in sequencing depth may have been influenced by the quality of the PCR products, the sequencing library, or some other factor(s). However, pursuing a conclusive cause of differences in valid read numbers is not easy. Therefore, this study focuses on the available data and only discusses the similarities and differences between the dominant bacterial phylogenetic groups and their indicator members. This study does not consider minor sequences that might be affected by the sequencing depth. We believe these approaches do not affect the overall interpretation of the results.

The lower sequencing depth may have caused lower detections at all taxonomic ranks in the Austrian samples. However, even though the sample size of Austria was smaller than Uganda, the identified OTUs from Austrian samples showed higher alpha-diversity in Table 5. The beta-diversity results, analyzed through PCA and hierarchical cluster analysis at the species and genus rank (Fig. 3 and Figure S7), clearly indicated a distinct separation between the regions of Austria and Uganda. However, the different sampling areas within Austria (A01 to A05) and a part of Uganda (U7 to U9) were not distinct in the ranks of family, order, class, and phylum (Figure S7). It shows that although the samples from the two sampling areas have similarities by cluster analysis at the ranks of family, order, class, and phylum, species compositions in microbiomes were partially different. This result is closely related to the distinctive characteristics of the bacterial community structure at both the genus and species levels in the two regions, that is, the presence of different dominant species leads to this result. It is important to note that although this method is considered reliable, utilizing complete 16S rRNA gene sequencing and genome sequencing may be necessary for future studies to further determine whether this result is entirely correct.

The region-specific differences in the composition of the lichen-associated bacterial microbiomes could result from isolation by distance mechanisms, i.e. by a geographic barrier resulting from the about 5430 km distance between the two sampling regions. On the other hand, the beta diversity may also have been affected by climatic differences between the two regions. Both regions are climatically classified as “alpine climate”, a categorization defined by monthly mean temperatures below 10 °C as part of group *E* in the Köppen climate classification [49]. Unlike other group *E* regions (polar and tundra with sparse precipitation), the Austrian and Ugandan sites receive relatively high annual precipitations of about 1300 mm and 2100 mm, respectively [50]. The sampling site elevations in Austria and Uganda were about 2600 m and 4700 m, respectively. From the elevations, air pressures are calculated as about 550 kPa and 720 kPa, respectively [51], which are not so different compared with the difference in the air temperature ranges. On the Rwenzori Mountains of equatorial Africa (Uganda), the modeled temperatures at an elevation of 4234 m ranged from − 0.1 °C to 10.9 °C with an annual mean of 4.9 °C [50]. On Mt. Brennkogel in the Eastern Alps (Austria), the modeled temperature at an elevation of 2191 m ranged from − 11.9 °C to 12.4 °C with an annual mean of − 0.3 °C [50]. The climate model shows a wider temperature range below and above the freezing point in the Eastern Alps area than in the Rwenzori Mountains area. Temperature ranges in the sampling seasons are 3 °C to 12 °C in July–August at the Austrian sites and 0 °C to 11 °C in February at the Ugandan sites [50], which are not so different compared with the annual temperature ranges. Therefore, differences in annual temperature ranges and the lower average temperature at the Austrian sampling sites may represent an important factor amongst the environmental variables that influenced the lichens and lichen-associated bacteria.

In conclusion, the region-specific differences in bacterial microbiome composition may result from one of three factors or a combination of those factors. These factors are (i) region-specific taxonomic differences between the lichen hosts, the algal symbionts might be a potentially important factor but further validation is required, (ii) an isolation by distance mechanism potentially restricting bacterial dispersal between the two alpine regions, and (iii) region-specific climatic differences favoring partially different sets of bacterial taxa sufficiently adapted to the local climatic conditions.

Based on the results of reference genomes, we have some speculations and hypotheses about the metabolic activities of bacteria in the two regions. For a given amount of error, a difference of 1% or less probably makes no difference when comparing geographically distant samples, therefore, In contrast to the revealed region-specific differences in the taxonomic composition of the lichen-associated bacterial microbiomes, relatively similar metabolic pathways were predicted for the microbiomes from the two distant regions. An important reason for the relatively similar pathways would be the host lichens belonging to the same species or even different species growing in the same ecotope. In other words, despite some differences in temperature ranges between the two regions, the high similarity of the predicted pathways could be related to the overall relatively similar alpine climates of the two regions. In other aspects, Metabolism is irrespective of acclimation and evolutionary adaptation and generally changes with temperature [52]. Freeze–thaw cycles likely occur more frequently in the Eastern Alps than in the seasonless Equatorial Rwenzori Mountains, which may cause the oxidation of biomolecules [53]. The PICRUSt 2.0 related higher frequencies of indicator OTUs in Austrian samples to glutathione metabolism on the KEGG Level 3 metabolic pathways (Fig. 6), suggesting more involvement of anti-oxidant activity of glutathione [54] in the East Alps microbiomes. However, the considerations are speculative. In the next phase of research, transcriptomic and metabolomic studies are required to obtain correct information. In addition, attention should be paid to genomes of closely related bacteria in which deletions can occur within one operon and this will lead to loss of expression of key enzymes. That is, the presence of a genetically predetermined process does not guarantee its occurrence in a particular strain or species.

It is important to note that the accuracy of PICRUSt-derived metabolic profiles relies on the availability, i.e. the presence/absence of reference genomes related to the identified OTUs in terms of their phylogenetic relationships. The bacteria inhabiting the lichens will need to be isolated and characterized to explicitly analyze and elaborate their characteristics of metabolic pathways or reconstruct and analyze bacterial genomes by culture-independent metagenomic approaches (e.g. bioinformatic analysis of metagenome-assembled genomes, MAGs). In addition, associated bacterial microbiomes may be directly or indirectly influenced by metabolisms, which also scaled with the temperature of the other partners in lichen symbiosis, i.e. fungal and algal/cyanobacterial partners. Careful selections are needed because the metabolic interactions of microbiomes of bacteria, as well as fungal and algal partners, may be different in lichens. The functional and ecological roles of bacterial microbiomes as the third components of the lichen symbiosis can be understood through the analyses based on OTUs, however, more data are necessary to contribute further to the advancement of research in this field.

### Supplementary Information

Below is the link to the electronic supplementary material.

Supplementary file1 (DOCX 6086 kb)
Supplementary Materials: Supplementary materials can be accessed and downloaded from the provided link: https://static-content.springer.com/esm/xxx. Table S1: Provided are the BioProject numbers, DRA accession numbers, and BioSample accession numbers associated with the sequence datasets of the V3-V4 region that have been deposited in the public DDBJ database; **Table S2**: Provided are the accession numbers for sequences of near-full-length fungal 18S rRNA gene originating from the examined rock tripe lichen samples. The table also includes corresponding lengths, the most closely associated species with their respective accession numbers and lengths, and the corresponding similarity values (%); **Table S3**: Provided are the accession numbers for fungal ITS sequences which were deleted partial 18S and partial 28S ribosomal RNA sequences originating from the examined rock tripe lichen samples. The table also includes corresponding lengths, the most closely associated species with their respective accession numbers and lengths, and the corresponding similarity values (%); **Table S4**: Provided are the accession numbers for fungal ITS sequences with partial 18S and partial 28S ribosomal RNA sequences originating from the examined rock tripe lichen samples. The table also includes corresponding lengths, the most closely associated species with their respective accession numbers and lengths, and the corresponding similarity values (%); **Table S5**: Overall outlook of S2-S3-S4 only with top-hit species names; **Table S6**: Information of *Umbilicaria aprina*, *U. rhizinata* and *U. africana* in the database of NCBI; **Table S7**: Provided are the accession numbers for sequences of near-full-length algal 18S rRNA gene originating from the examined rock tripe lichen samples. The table also includes corresponding lengths, the most closely associated species with their respective accession numbers, and the corresponding similarity values (%).Figure S1: Photographs of the thalli taken at the collection sites on Mt. Brennkogel in the Eastern Alps (Austria) and the Rwenzori Mountains (Uganda).; **Figure S2**: Illustrated the rarefaction curves derived from the read and OTU counts of 5 sampling sites on Mt. Brennkogel in the Eastern Alps (Austria) and 11 sampling sites on the Rwenzori Mountains (Uganda); **Figure S3**: The distribution of bacterial classes among OTUs in lichen samples collected from the Eastern Alps (Austria, A01 to A05) and the Rwenzori Mountains (Uganda, U1 to U11) was examined; **Figure S4**: The distribution of bacterial orders among OTUs in lichen samples collected from the Eastern Alps (Austria, A01 to A05) and the Rwenzori Mountains (Uganda, U1 to U11) was examined; **Figure S5**: The distribution of bacterial families among OTUs in lichen samples collected from the Eastern Alps (Austria, A01 to A05) and the Rwenzori Mountains (Uganda, U1 to U11) was examined; **Figure S6**: The distribution of bacterial genera among OTUs in lichen samples collected from the Eastern Alps (Austria, A01 to A05) and the Rwenzori Mountains (Uganda, U1 to U11) was examined; **Figure S7**: PCA plots were generated to visualize the distribution of OTU-derived species (top left), genera (top middle), families (top right), orders (bottom left), classes (bottom middle), and phyla (bottom right) among lichen samples collected from Mt. Stanley of the Eastern Alps (Austria, denoted in red) and the Rwenzori Mountains (Uganda, denoted in green); **Figure S8**: Statistically significant disparities (*P* < 0.05) in the relative abundances of indicator OTUs were assessed using ANCOM-BC between the Eastern Alps' Mt. Brennkogel (denoted in red) and the Rwenzori Mountains' Mt. Stanley (denoted in green) The names of potentially associated genera or phyla, if applicable, are displayed beneath the respective OTU identifiers; **Figure S9**: Metabolic pathways at KEGG Level 1 identified within the indicator OTUs originating from the Eastern Alps' Mt. Brennkogel (denoted in red) and the Rwenzori Mountains' Mt. Stanley (denoted in green); Figure S1**0**: Metabolic pathways at KEGG Level 2 identified within the indicator OTUs originating from the Eastern Alps' Mt. Brennkogel (denoted in red) and the Rwenzori Mountains' Mt. Stanley (denoted in green); Figure S1**1**: Metabolic pathways at KEGG Level 3 identified within the indicator OTUs originating from the Eastern Alps' Mt. Brennkogel (denoted in red) and the Rwenzori Mountains' Mt. Stanley (denoted in green).

## Data Availability

The DDBJ Sequence Read Archive (DRA014939) contains the raw sequence data for the Austria samples, while the associated project data can be found under BioProject (PRJDB14357), and the sample data are accessible via BioSample (SAMD00547134 to SAMD00547138). The DDBJ Sequence Read Archive (DRA014883) contains the raw sequence data for the Uganda samples, while the associated project data can be found under BioProject (PRJDB14357), and the sample data are accessible via BioSample (SAMD00535656 to SAMD00535666).
