# Supplementary material for: Microbiomic Analysis of Bacteria Associated with Rock Tripe Lichens from Alpine Areas in Eastern Alps and Equatorial Africa
Source: Curr Microbiol. 2024 Mar 14;81(5):115. doi: 10.1007/s00284-024-03626-8 (PMC10940493; doi:10.1007/s00284-024-03626-8)

Supplementary materials (Tables S1 to S7, and Figures S1 to S11) of

“Microbiomc Analysis of Bacteria Associated with Rock Tripe Lichens from Alpine Areas in Eastern Alps and Equatorial Africa”

Zichen He

Graduate School of Integrated Science for Life, Hiroshima University

Higashi-hiroshima 739-8528, Japan

[szichenhe@gmail.com](mailto:szichenhe@gmail.com); ORCID 0009-0001-9402-1210

Takeshi Naganuma

Corresponding author

Graduate School of Integrated Science for Life, Hiroshima University

Higashi-hiroshima 739-8528, Japan

[takn@hiroshima-u.ac.jp](mailto:takn@hiroshima-u.ac.jp); ORCID 0000-0003-1925-9461; +81-82-424-7986

Ryosuke Nakai

Bioproduction Research Institute, National Institute of Advanced Industrial Science and Technology

Sapporo 062-8517, Japan

[nakai-ryosuke@aist.go.jp](mailto:nakai-ryosuke@aist.go.jp); ORCID 0000-0002-3078-6695

Jun Uetake

Field Science Center for Northern Biosphere, Hokkaido University

Sapporo 060-0811, Japan

[jun.uetake@fsc.hokudai.ac.jp](mailto:jun.uetake@fsc.hokudai.ac.jp); ORCID 0000-0001-8135-2531

Martin W. Hahn

Research Department for Limnology, Universität Innsbruck

Mondsee A-5310, Austria

[martin.hahn@uibk.ac.at](mailto:martin.hahn@uibk.ac.at); ORCID 0000-0003-0501-2556

**Corresponding author**: Takeshi Naganuma, [takn@hiroshima-u.ac.jp](mailto:takn@hiroshima-u.ac.jp), ORCID 0000-0003-1925-9461, +81-82-424-7986

Table S1. Provided are the BioProject numbers, DRA accession numbers, and BioSample accession numbers associated with the sequence datasets of the V3-V4 region that have been deposited in the public DDBJ database.

| Sample | BioProject # | DRA acc. # | BioSample acc. # |
| --- | --- | --- | --- |
| A01 | PRJDB14357 | DRA014939 | SAMD00547134 |
| A02 |  |  | SAMD00547135 |
| A03 |  |  | SAMD00547136 |
| A04 |  |  | SAMD00547137 |
| A05 |  |  | SAMD00547138 |
| U1 |  | DRA014883 | SAMD00535656 |
| U2 |  |  | SAMD00535657 |
| U3 |  |  | SAMD00535658 |
| U4 |  |  | SAMD00535659 |
| U5 |  |  | SAMD00535660 |
| U6 |  |  | SAMD00535661 |
| U7 |  |  | SAMD00535662 |
| U8 |  |  | SAMD00535663 |
| U9 |  |  | SAMD00535664 |
| U10 |  |  | SAMD00535665 |
| U11 |  |  | SAMD00535666 |

Table S2. Provided are the accession numbers for sequences of near-full-length fungal 18S rRNA gene originating from the examined rock tripe lichen samples. The table also includes corresponding lengths, the most closely associated species with their respective accession numbers and lengths, and the corresponding similarity values (%).

| Sample | Accession # | Length | Closest species | Accession # | Length | Similarity (%) |
| --- | --- | --- | --- | --- | --- | --- |
| A01 | LC730222 | 1731 | *Umbilicaria aprina* voucher agrED360 | KY948014 | 2217 | 99.88 |
|  |  |  | *Umbilicaria rhizinata* voucher agrED295 | KY948011 | 2303 | 99.88 |
| A02 | LC730223 | 1729 | *Umbilicaria aprina* voucher agrED360 | KY948014 | 2217 | 99.71 |
|  |  |  | *Umbilicaria rhizinata* voucher agrED295 | KY948011 | 2303 | 99.71 |
| A03 | LC730224 | 1728 | *Umbilicaria aprina* voucher agrED360 | KY948014 | 2217 | 99.94 |
|  |  |  | *Umbilicaria rhizinata* voucher agrED295 | KY948011 | 2303 | 99.88 |
| A04 | LC730225 | 1732 | *Umbilicaria aprina* voucher agrED360 | KY948014 | 2217 | 99.88 |
|  |  |  | *Umbilicaria rhizinata* voucher agrED295 | KY948011 | 2303 | 99.88 |
| A05 | LC730226 | 1727 | *Umbilicaria aprina* voucher agrED360 | KY948014 | 2217 | 99.35 |
|  |  |  | *Umbilicaria rhizinata* voucher agrED295 | KY948011 | 2303 | 99.30 |
| U1 | LC730211 | 1726 | *Umbilicaria aprina* voucher agrED360 | KY948014 | 2217 | 99.59 |
|  |  |  | *Umbilicaria rhizinata* voucher agrED295 | KY948011 | 2303 | 99.59 |
| U2 | LC730212 | 1724 | *Umbilicaria aprina* voucher agrED360 | KY948014 | 2217 | 99.65 |
|  |  |  | *Umbilicaria rhizinata* voucher agrED295 | KY948011 | 2303 | 99.65 |
| U3 | LC730213 | 1725 | *Umbilicaria aprina* voucher agrED360 | KY948014 | 2217 | 99.76 |
|  |  |  | *Umbilicaria rhizinata* voucher agrED295 | KY948011 | 2303 | 99.77 |
| U4 | LC730214 | 1728 | *Umbilicaria aprina* voucher agrED360 | KY948014 | 2217 | 99.65 |
|  |  |  | *Umbilicaria rhizinata* voucher agrED295 | KY948011 | 2303 | 99.65 |
| U5 | LC730215 | 1728 | *Umbilicaria aprina* voucher agrED360 | KY948014 | 2217 | 99.76 |
|  |  |  | *Umbilicaria rhizinata* voucher agrED295 | KY948011 | 2303 | 99.77 |
| U6 | LC730216 | 1724 | *Umbilicaria aprina* voucher agrED360 | KY948014 | 2217 | 99.76 |
|  |  |  | *Umbilicaria rhizinata* voucher agrED295 | KY948011 | 2303 | 99.77 |
| U7 | LC730217 | 1725 | *Umbilicaria aprina* voucher agrED360 | KY948014 | 2217 | 99.88 |
|  |  |  | *Umbilicaria rhizinata* voucher agrED295 | KY948011 | 2303 | 99.88 |
| U8 | LC730218 | 1727 | *Umbilicaria aprina* voucher agrED360 | KY948014 | 2217 | 99.53 |
|  |  |  | *Umbilicaria rhizinata* voucher agrED295 | KY948011 | 2303 | 99.83 |
| U9 | LC730219 | 1730 | *Umbilicaria aprina* voucher agrED360 | KY948014 | 2217 | 99.82 |
|  |  |  | *Umbilicaria rhizinata* voucher agrED295 | KY948011 | 2303 | 99.82 |
| U10 | LC730220 | 1730 | *Umbilicaria aprina* voucher agrED360 | KY948014 | 2217 | 99.65 |
|  |  |  | *Umbilicaria rhizinata* voucher agrED295 | KY948011 | 2303 | 99.65 |
| U11 | LC730221 | 1735 | *Umbilicaria aprina* voucher agrED360 | KY948014 | 2217 | 99.76 |
|  |  |  | *Umbilicaria rhizinata* voucher agrED295 | KY948011 | 2303 | 99.77 |

Table S3. Provided are the accession numbers for fungal ITS sequences which were deleted partial 18S and partial 28S ribosomal RNA sequences originating from the examined rock tripe lichen samples. The table also includes corresponding lengths, the most closely associated species with their respective accession numbers and lengths, and the corresponding similarity values (%).

| **Notice 1**: All the sequences are the same | | |  |  |  |  |
| --- | --- | --- | --- | --- | --- | --- |
| **Notice 2**: ITS sequences of *Umbilicaria spp.* are around 485 bp | | | |  |  |  |
| Sample | Accession # | Length | Closest species | Accession # | Length | Similarity (%) |
| A01 | LC744762 | 485 | *Umbilicaria africana* voucher acpED473 | KY947743 | 1138 | 99.79 |
|  |  |  | *Umbilicaria aprina* isolate AFTOL-ID 7153 | HM161483 | 555 | 99.79 |
|  |  |  | *Umbilicaria aprina* isolate AFTOL-ID 7116 | HM161502 | 572 | 99.79 |
| A02 | LC744763 | 485 | *Umbilicaria africana* voucher acpED473 | KY947743 | 1138 | 99.79 |
|  |  |  | *Umbilicaria aprina* isolate AFTOL-ID 7153 | HM161483 | 555 | 99.79 |
|  |  |  | *Umbilicaria aprina* isolate AFTOL-ID 7116 | HM161502 | 572 | 99.79 |
| A03 | LC744764 | 485 | *Umbilicaria africana* voucher acpED473 | KY947743 | 1138 | 99.79 |
|  |  |  | *Umbilicaria aprina* isolate AFTOL-ID 7153 | HM161483 | 555 | 99.79 |
|  |  |  | *Umbilicaria aprina* isolate AFTOL-ID 7116 | HM161502 | 572 | 99.79 |
| A04 | LC744765 | 485 | *Umbilicaria africana* voucher acpED473 | KY947743 | 1138 | 99.79 |
|  |  |  | *Umbilicaria aprina* isolate AFTOL-ID 7153 | HM161483 | 555 | 99.79 |
|  |  |  | *Umbilicaria aprina* isolate AFTOL-ID 7116 | HM161502 | 572 | 99.79 |
| A05 | LC744766 | 485 | *Umbilicaria africana* voucher acpED473 | KY947743 | 1138 | 99.79 |
|  |  |  | *Umbilicaria aprina* isolate AFTOL-ID 7153 | HM161483 | 555 | 99.79 |
|  |  |  | *Umbilicaria aprina* isolate AFTOL-ID 7116 | HM161502 | 572 | 99.79 |
| U1 | LC744767 | 485 | *Umbilicaria africana* voucher acpED473 | KY947743 | 1138 | 99.79 |
|  |  |  | *Umbilicaria aprina* isolate AFTOL-ID 7153 | HM161483 | 555 | 99.79 |
|  |  |  | *Umbilicaria aprina* isolate AFTOL-ID 7116 | HM161502 | 572 | 99.79 |
| U2 | LC744768 | 485 | *Umbilicaria africana* voucher acpED473 | KY947743 | 1138 | 99.79 |
|  |  |  | *Umbilicaria aprina* isolate AFTOL-ID 7153 | HM161483 | 555 | 99.79 |
|  |  |  | *Umbilicaria aprina* isolate AFTOL-ID 7116 | HM161502 | 572 | 99.79 |
| U3 | LC744769 | 485 | *Umbilicaria africana* voucher acpED473 | KY947743 | 1138 | 99.79 |
|  |  |  | *Umbilicaria aprina* isolate AFTOL-ID 7153 | HM161483 | 555 | 99.79 |
|  |  |  | *Umbilicaria aprina* isolate AFTOL-ID 7116 | HM161502 | 572 | 99.79 |
| U4 | LC744770 | 485 | *Umbilicaria africana* voucher acpED473 | KY947743 | 1138 | 99.79 |
|  |  |  | *Umbilicaria aprina* isolate AFTOL-ID 7153 | HM161483 | 555 | 99.79 |
|  |  |  | *Umbilicaria aprina* isolate AFTOL-ID 7116 | HM161502 | 572 | 99.79 |
| U5 | LC744771 | 485 | *Umbilicaria africana* voucher acpED473 | KY947743 | 1138 | 99.79 |
|  |  |  | *Umbilicaria aprina* isolate AFTOL-ID 7153 | HM161483 | 555 | 99.79 |
|  |  |  | *Umbilicaria aprina* isolate AFTOL-ID 7116 | HM161502 | 572 | 99.79 |
| U6 | LC744772 | 485 | *Umbilicaria africana* voucher acpED473 | KY947743 | 1138 | 99.79 |
|  |  |  | *Umbilicaria aprina* isolate AFTOL-ID 7153 | HM161483 | 555 | 99.79 |
|  |  |  | *Umbilicaria aprina* isolate AFTOL-ID 7116 | HM161502 | 572 | 99.79 |
| U7 | LC744773 | 485 | *Umbilicaria africana* voucher acpED473 | KY947743 | 1138 | 99.79 |
|  |  |  | *Umbilicaria aprina* isolate AFTOL-ID 7153 | HM161483 | 555 | 99.79 |
|  |  |  | *Umbilicaria aprina* isolate AFTOL-ID 7116 | HM161502 | 572 | 99.79 |
| U8 | LC744774 | 485 | *Umbilicaria africana* voucher acpED473 | KY947743 | 1138 | 99.79 |
|  |  |  | *Umbilicaria aprina* isolate AFTOL-ID 7153 | HM161483 | 555 | 99.79 |
|  |  |  | *Umbilicaria aprina* isolate AFTOL-ID 7116 | HM161502 | 572 | 99.79 |
| U9 | LC744775 | 485 | *Umbilicaria africana* voucher acpED473 | KY947743 | 1138 | 99.79 |
|  |  |  | *Umbilicaria aprina* isolate AFTOL-ID 7153 | HM161483 | 555 | 99.79 |
|  |  |  | *Umbilicaria aprina* isolate AFTOL-ID 7116 | HM161502 | 572 | 99.79 |
| U10 | LC744776 | 485 | *Umbilicaria africana* voucher acpED473 | KY947743 | 1138 | 99.79 |
|  |  |  | *Umbilicaria aprina* isolate AFTOL-ID 7153 | HM161483 | 555 | 99.79 |
|  |  |  | *Umbilicaria aprina* isolate AFTOL-ID 7116 | HM161502 | 572 | 99.79 |
| U11 | LC744777 | 485 | *Umbilicaria africana* voucher acpED473 | KY947743 | 1138 | 99.79 |
|  |  |  | *Umbilicaria aprina* isolate AFTOL-ID 7153 | HM161483 | 555 | 99.79 |
|  |  |  | *Umbilicaria aprina* isolate AFTOL-ID 7116 | HM161502 | 572 | 99.79 |

Table S4. Provided are the accession numbers for fungal ITS sequences with partial 18S and partial 28S ribosomal RNA sequences originating from the examined rock tripe lichen samples. The table also includes corresponding lengths, the most closely associated species with their respective accession numbers and lengths, and the corresponding similarity values (%).

| Sample | Accession # | Length | Closest species | Accession # | Length | Similarity (%) |
| --- | --- | --- | --- | --- | --- | --- |
| A01 | LC744762 | 544 | *Umbilicaria aprina* isolate AFTOL-ID 7116 | HM161502 | 572 | 99.25 |
|  |  |  | *Umbilicaria africana* voucher acpED473 | KY947743 | 1138 | 99.24 |
|  |  |  | *Umbilicaria aprina* isolate AFTOL-ID 7153 | HM161483 | 555 | 99.06 |
| A02 | LC744763 | 547 | *Umbilicaria aprina* isolate AFTOL-ID 7116 | HM161502 | 572 | 99.44 |
|  |  |  | *Umbilicaria aprina* isolate AFTOL-ID 7153 | HM161483 | 555 | 99.26 |
|  |  |  | *Umbilicaria africana* voucher acpED473 | KY947743 | 1138 | 99.06 |
| A03 | LC744764 | 546 | *Umbilicaria aprina* isolate AFTOL-ID 7116 | HM161502 | 572 | 99.25 |
|  |  |  | *Umbilicaria africana* voucher acpED473 | KY947743 | 1138 | 99.25 |
|  |  |  | *Umbilicaria aprina* isolate AFTOL-ID 7153 | HM161483 | 555 | 99.07 |
| A04 | LC744765 | 545 | *Umbilicaria aprina* isolate AFTOL-ID 7116 | HM161502 | 572 | 99.44 |
|  |  |  | *Umbilicaria aprina* isolate AFTOL-ID 7153 | HM161483 | 555 | 99.25 |
|  |  |  | *Umbilicaria africana* voucher acpED473 | KY947743 | 1138 | 99.06 |
| A05 | LC744766 | 544 | *Umbilicaria aprina* isolate AFTOL-ID 7116 | HM161502 | 572 | 99.25 |
|  |  |  | *Umbilicaria africana* voucher acpED473 | KY947743 | 1138 | 99.25 |
|  |  |  | *Umbilicaria aprina* isolate AFTOL-ID 7153 | HM161483 | 555 | 99.07 |
| U1 | LC744767 | 542 | *Umbilicaria africana* voucher acpED473 | KY947743 | 1138 | 99.43 |
|  |  |  | *Umbilicaria aprina* isolate AFTOL-ID 7116 | HM161502 | 572 | 99.05 |
|  |  |  | *Umbilicaria aprina* isolate AFTOL-ID 7153 | HM161483 | 555 | 98.87 |
| U2 | LC744768 | 543 | *Umbilicaria africana* voucher acpED473 | KY947743 | 1138 | 99.43 |
|  |  |  | *Umbilicaria aprina* isolate AFTOL-ID 7116 | HM161502 | 572 | 99.05 |
|  |  |  | *Umbilicaria aprina* isolate AFTOL-ID 7153 | HM161483 | 555 | 98.87 |
| U3 | LC744769 | 544 | *Umbilicaria aprina* isolate AFTOL-ID 7153 | HM161483 | 555 | 99.25 |
|  |  |  | *Umbilicaria aprina* isolate AFTOL-ID 7116 | HM161502 | 572 | 99.25 |
|  |  |  | *Umbilicaria africana* voucher acpED473 | KY947743 | 1138 | 99.25 |
| U4 | LC744770 | 544 | *Umbilicaria aprina* isolate AFTOL-ID 7153 | HM161483 | 555 | 99.25 |
|  |  |  | *Umbilicaria aprina* isolate AFTOL-ID 7116 | HM161502 | 572 | 99.25 |
|  |  |  | *Umbilicaria africana* voucher acpED473 | KY947743 | 1138 | 99.25 |
| U5 | LC744771 | 545 | *Umbilicaria aprina* isolate AFTOL-ID 7153 | HM161483 | 555 | 98.89 |
|  |  |  | *Umbilicaria aprina* isolate AFTOL-ID 7116 | HM161502 | 572 | 98.89 |
|  |  |  | *Umbilicaria africana* voucher acpED473 | KY947743 | 1138 | 98.52 |
| U6 | LC744772 | 546 | *Umbilicaria aprina* isolate AFTOL-ID 7153 | HM161483 | 555 | 98.70 |
|  |  |  | *Umbilicaria aprina* isolate AFTOL-ID 7116 | HM161502 | 572 | 98.70 |
|  |  |  | *Umbilicaria africana* voucher acpED473 | KY947743 | 1138 | 98.70 |
| U7 | LC744773 | 543 | *Umbilicaria aprina* isolate AFTOL-ID 7153 | HM161483 | 555 | 99.44 |
|  |  |  | *Umbilicaria aprina* isolate AFTOL-ID 7116 | HM161502 | 572 | 99.44 |
|  |  |  | *Umbilicaria africana* voucher acpED473 | KY947743 | 1138 | 99.06 |
| U8 | LC744774 | 546 | *Umbilicaria aprina* isolate AFTOL-ID 7153 | HM161483 | 555 | 99.25 |
|  |  |  | *Umbilicaria aprina* isolate AFTOL-ID 7116 | HM161502 | 572 | 99.25 |
|  |  |  | *Umbilicaria africana* voucher acpED473 | KY947743 | 1138 | 98.87 |
| U9 | LC744775 | 545 | *Umbilicaria aprina* isolate AFTOL-ID 7153 | HM161483 | 555 | 99.24 |
|  |  |  | *Umbilicaria aprina* isolate AFTOL-ID 7116 | HM161502 | 572 | 99.24 |
|  |  |  | *Umbilicaria africana* voucher acpED473 | KY947743 | 1138 | 99.24 |
| U10 | LC744776 | 544 | *Umbilicaria africana* voucher acpED473 | KY947743 | 1138 | 99.43 |
|  |  |  | *Umbilicaria aprina* isolate AFTOL-ID 7153 | HM161483 | 555 | 99.05 |
|  |  |  | *Umbilicaria aprina* isolate AFTOL-ID 7116 | HM161502 | 572 | 99.05 |
| U11 | LC744777 | 543 | *Umbilicaria aprina* isolate AFTOL-ID 7153 | HM161483 | 555 | 99.24 |
|  |  |  | *Umbilicaria aprina* isolate AFTOL-ID 7116 | HM161502 | 572 | 99.24 |
|  |  |  | *Umbilicaria africana* voucher acpED473 | KY947743 | 1138 | 99.24 |

Table S5. The overall outlook of S2-S3-S4 only with top-hit species names

| Sample | Closest species of 18S sequence | Closest species of only ITS sequence | Closest species of ITS with partial 18S and partial 28S sequence |
| --- | --- | --- | --- |
| A01 | *Umbilicaria rhizinata* voucher agrED295 | *Umbilicaria africana* voucher acpED473 | *Umbilicaria aprina* isolate AFTOL-ID 7116 |
| A02 | *Umbilicaria rhizinata* voucher agrED295 | *Umbilicaria africana* voucher acpED473 | *Umbilicaria aprina* isolate AFTOL-ID 7116 |
| A03 | *Umbilicaria aprina* voucher agrED360 | *Umbilicaria africana* voucher acpED473 | *Umbilicaria aprina* isolate AFTOL-ID 7116 |
| A04 | *Umbilicaria rhizinata* voucher agrED295 | *Umbilicaria africana* voucher acpED473 | *Umbilicaria aprina* isolate AFTOL-ID 7116 |
| A05 | *Umbilicaria aprina* voucher agrED360 | *Umbilicaria africana* voucher acpED473 | *Umbilicaria aprina* isolate AFTOL-ID 7116 |
| U1 | *Umbilicaria rhizinata* voucher agrED295 | *Umbilicaria africana* voucher acpED473 | *Umbilicaria africana* voucher acpED473 |
| U2 | *Umbilicaria rhizinata* voucher agrED295 | *Umbilicaria africana* voucher acpED473 | *Umbilicaria africana* voucher acpED473 |
| U3 | *Umbilicaria rhizinata* voucher agrED295 | *Umbilicaria africana* voucher acpED473 | *Umbilicaria aprina* isolate AFTOL-ID 7153 |
| U4 | *Umbilicaria rhizinata* voucher agrED295 | *Umbilicaria africana* voucher acpED473 | *Umbilicaria aprina* isolate AFTOL-ID 7153 |
| U5 | *Umbilicaria rhizinata* voucher agrED295 | *Umbilicaria africana* voucher acpED473 | *Umbilicaria aprina* isolate AFTOL-ID 7153 |
| U6 | *Umbilicaria rhizinata* voucher agrED295 | *Umbilicaria africana* voucher acpED473 | *Umbilicaria aprina* isolate AFTOL-ID 7153 |
| U7 | *Umbilicaria rhizinata* voucher agrED295 | *Umbilicaria africana* voucher acpED473 | *Umbilicaria aprina* isolate AFTOL-ID 7153 |
| U8 | *Umbilicaria rhizinata* voucher agrED295 | *Umbilicaria africana* voucher acpED473 | *Umbilicaria aprina* isolate AFTOL-ID 7153 |
| U9 | *Umbilicaria rhizinata* voucher agrED295 | *Umbilicaria africana* voucher acpED473 | *Umbilicaria aprina* isolate AFTOL-ID 7153 |
| U10 | *Umbilicaria rhizinata* voucher agrED295 | *Umbilicaria africana* voucher acpED473 | *Umbilicaria africana* voucher acpED473 |
| U11 | *Umbilicaria rhizinata* voucher agrED295 | *Umbilicaria africana* voucher acpED473 | *Umbilicaria aprina* isolate AFTOL-ID 7153 |

Table S6. Information on *Umbilicaria aprina*, *U. rhizinata,* and *U. africana* in the database of NCBI

| Species name | Total sequences amount | Only 18S sequences amount | Only ITS sequences amount | Near full-length 18S with ITS sequences amount |
| --- | --- | --- | --- | --- |
| *Umbilicaria aprina* | 85 | 1 | 39 | 1 |
| *U. rhizinata* | 3 | 0 | 0 | 1 |
| *U. africana* | 8 | 0 | 3 | 0 |

Table S7. Provided are the accession numbers for sequences of near-full-length algal 18S rRNA gene originating from the examined rock tripe lichen samples. The table also includes corresponding lengths, the most closely associated species with their respective accession numbers, and the corresponding similarity values (%).

| Sample | <Accession # | Closest species | Accession # | Similarity (%) |
| --- | --- | --- | --- | --- |
| A01 | LC730238 | *Trebouxia jamesii* (UBT-86.132E2) | Z68700 | 96.89 |
| A02 | LC730239 | *Trebouxia jamesii* (UBT-86.132E2) | Z68700 | 96.85 |
| A03 | LC730240 | *Trebouxia jamesii* (UBT-86.132E2) | Z68700 | 97.67 |
| A04 | LC730241 | *Trebouxia jamesii* (UBT-86.132E2) | Z68700 | 97.02 |
| A05 | LC730242 | *Trebouxia jamesii* (UBT-86.132E2) | Z68700 | 97.37 |
| U1 | LC730227 | *Trebouxia jamesii* (UBT-86.132E2) | Z68700 | 99.16 |
| U2 | LC730228 | *Trebouxia jamesii* (UBT-86.132E2) | Z68700 | 98.46 |
| U3 | LC730229 | *Trebouxia jamesii* (UBT-86.132E2) | Z68700 | 98.63 |
| U4 | LC730230 | *Trebouxia jamesii* (UBT-86.132E2) | Z68700 | 99.04 |
| U5 | LC730231 | *Trebouxia jamesii* (UBT-86.132E2) | Z68700 | 99.23 |
| U6 | LC730232 | *Trebouxia jamesii* (UBT-86.132E2) | Z68700 | 99.40 |
| U7 | LC730233 | *Trebouxia jamesii* (UBT-86.132E2) | Z68700 | 99.35 |
| U8 | LC730234 | *Trebouxia jamesii* (UBT-86.132E2) | Z68700 | 99.23 |
| U9 | LC730235 | *Trebouxia jamesii* (UBT-86.132E2) | Z68700 | 99.28 |
| U10 | LC730236 | *Trebouxia jamesii* (UBT-86.132E2) | Z68700 | 99.46 |
| U11 | LC730237 | *Trebouxia jamesii* (UBT-86.132E2) | Z68700 | 99.46 |


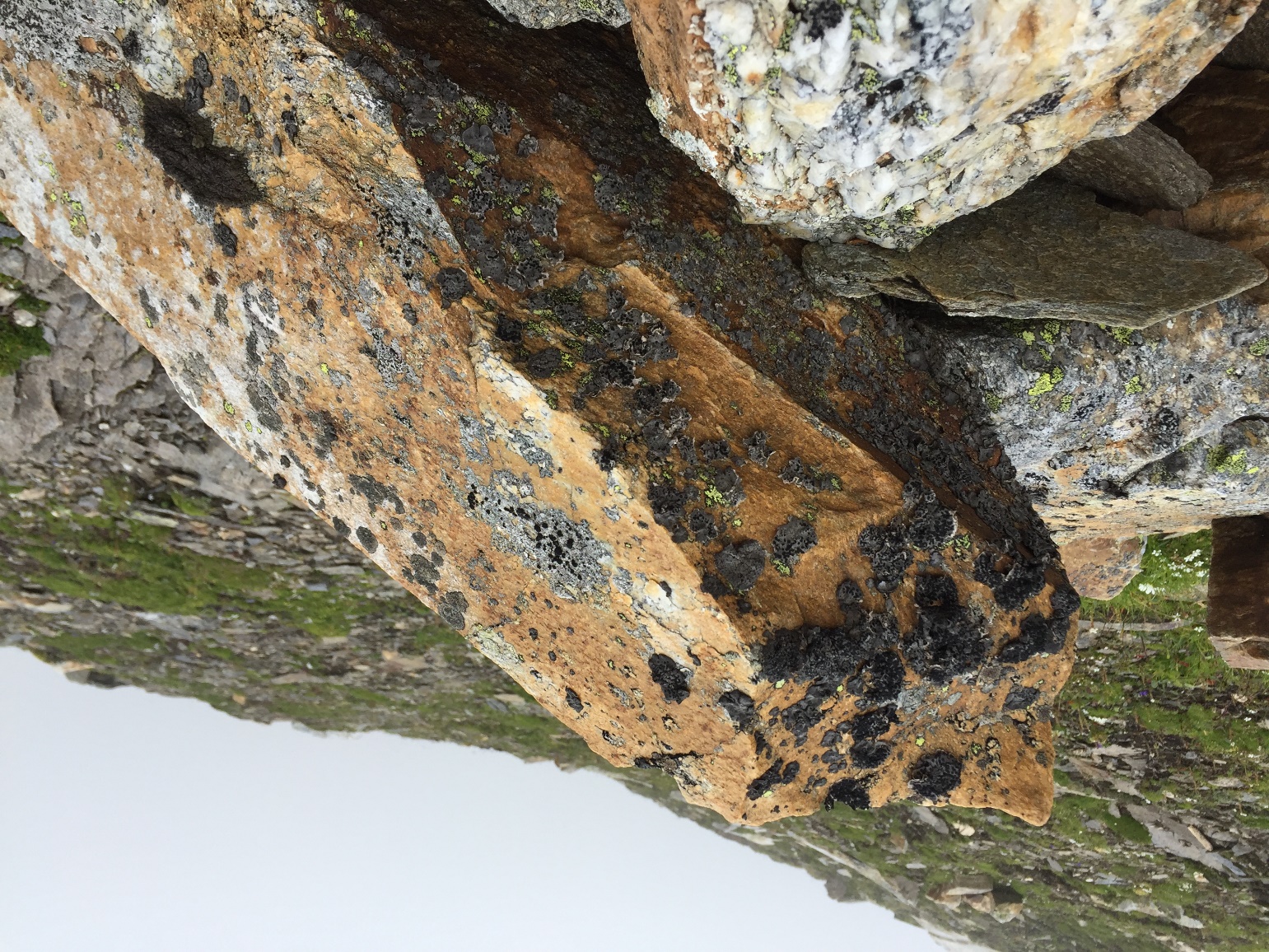


**(a1)**


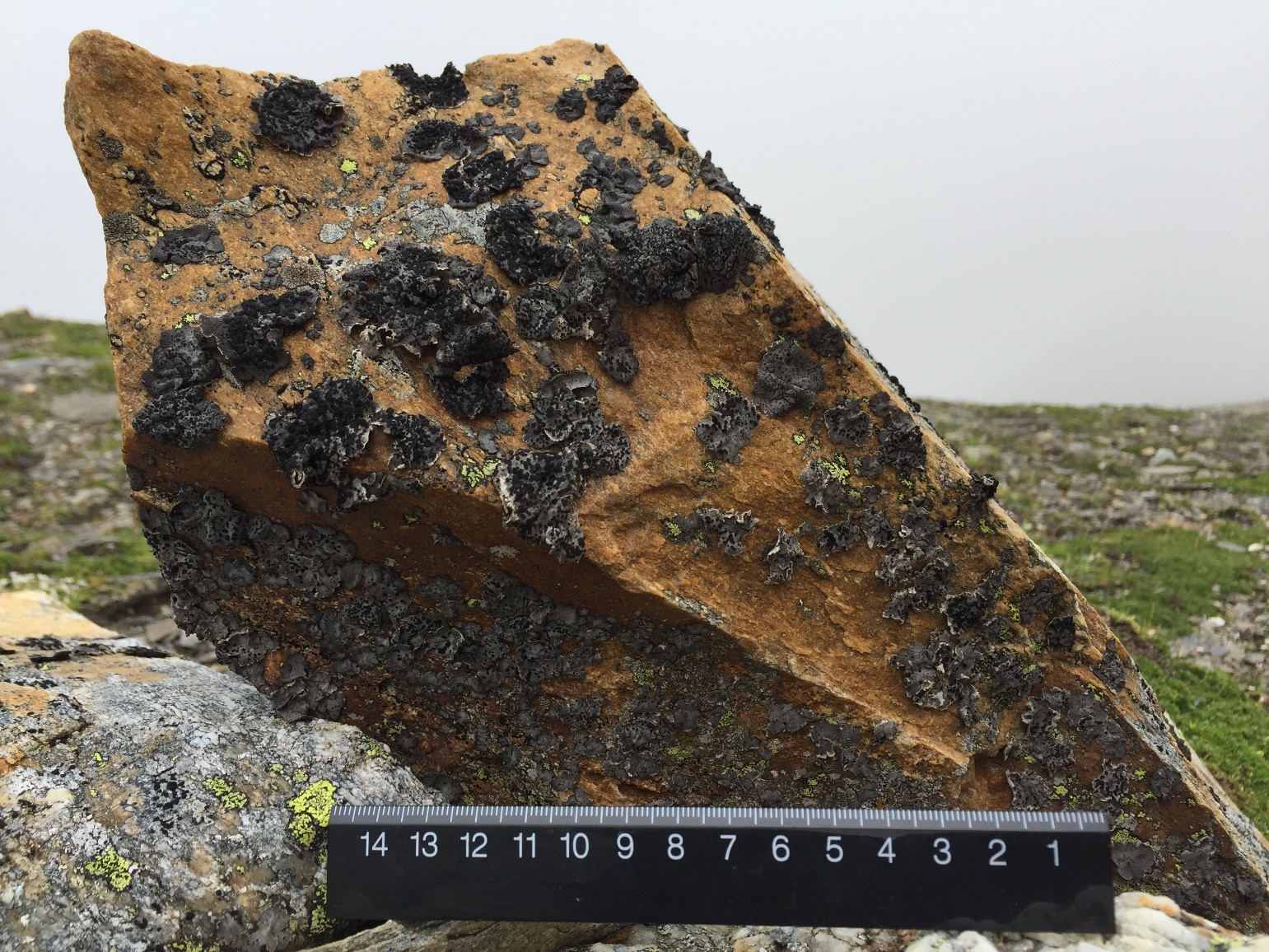


**(a2)**


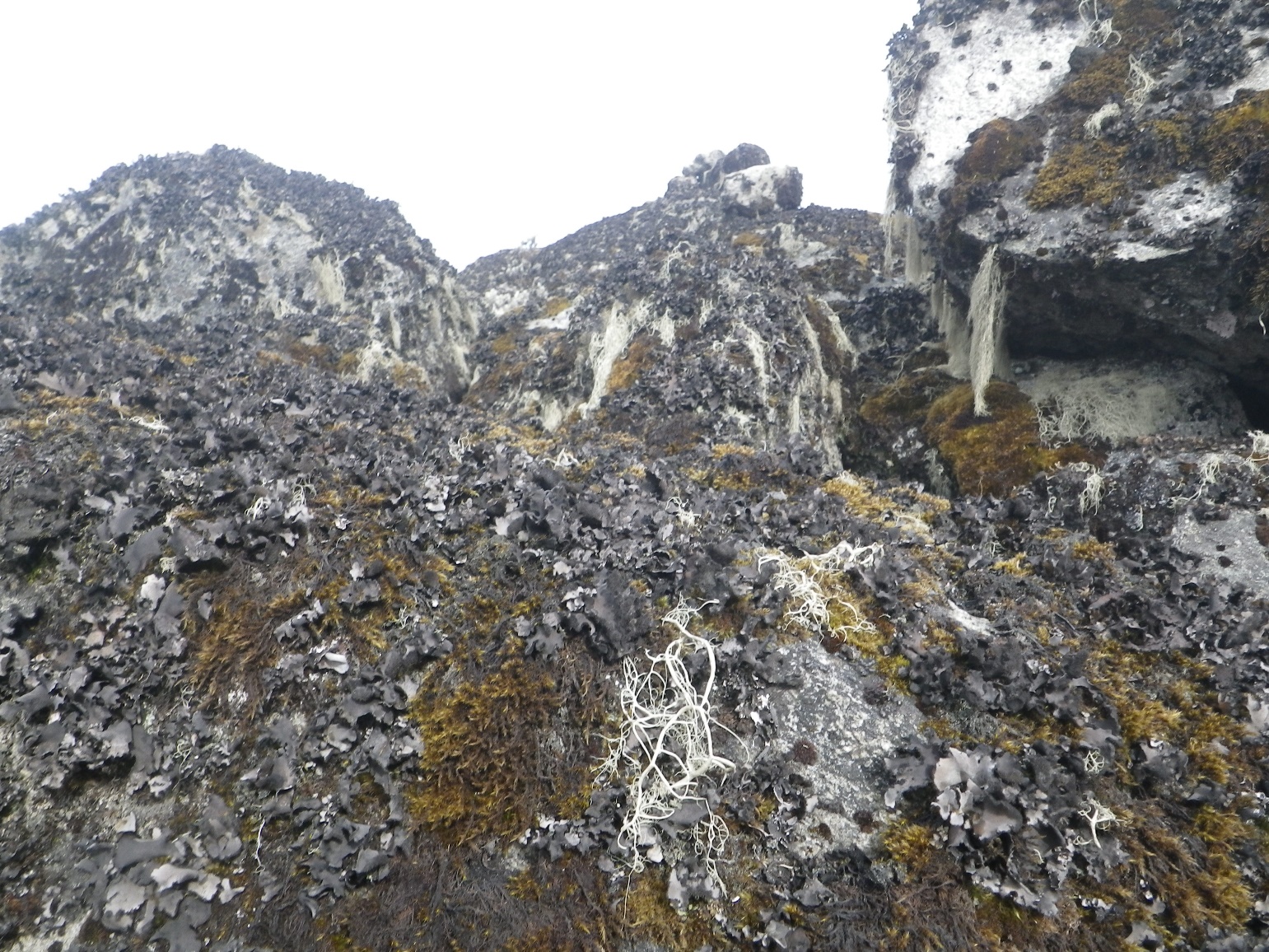


**(b1)**


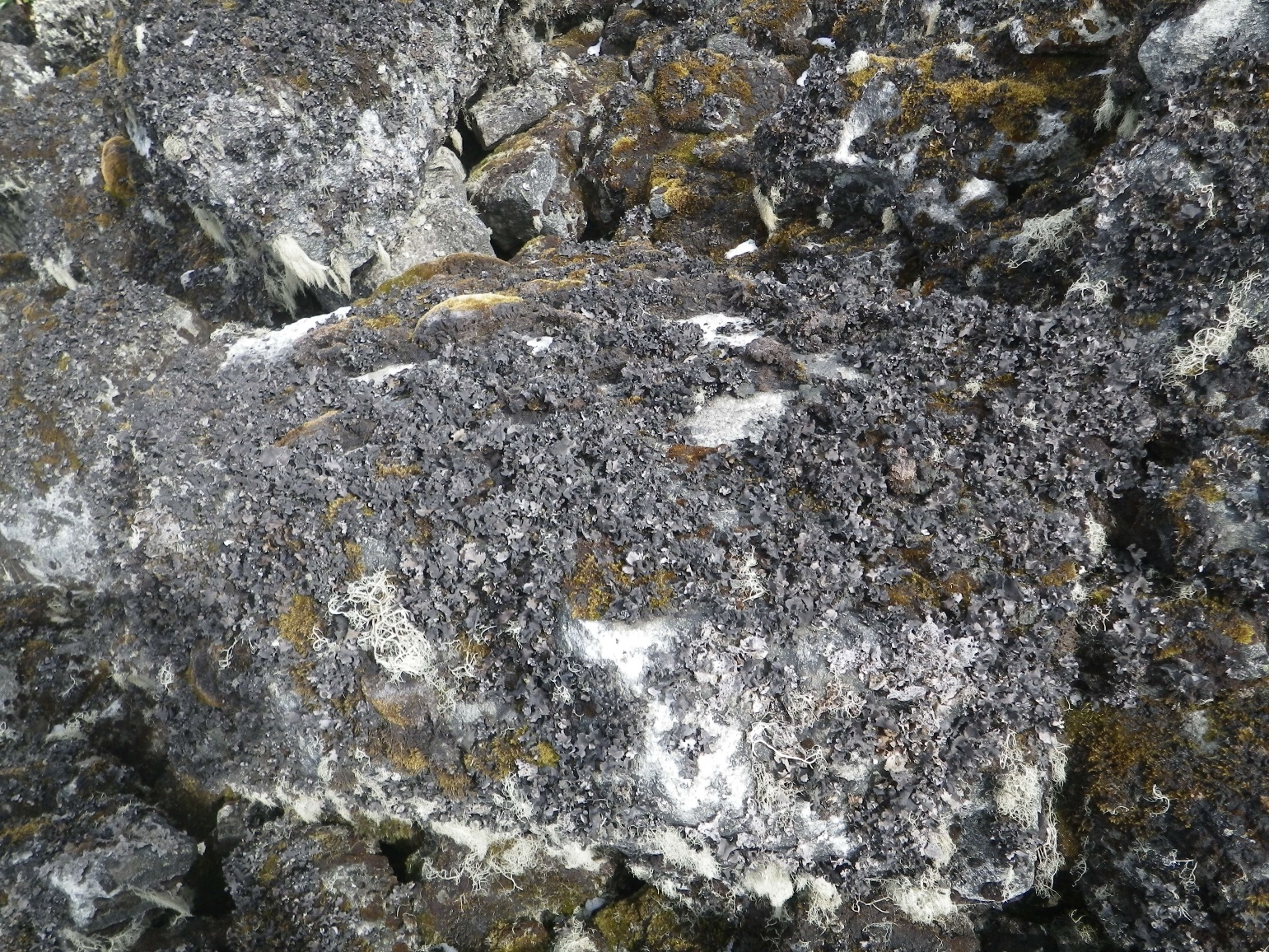


**(b2)**

Figure S1. Photographs of the thalli taken at the collection sites. **(a1), (a2),** Mt. Brennkogel in the Eastern Alps (Austria). **(b1), (b2),** The Rwenzori Mountains (Uganda). Only *Umbilicaria* thalli were collected.


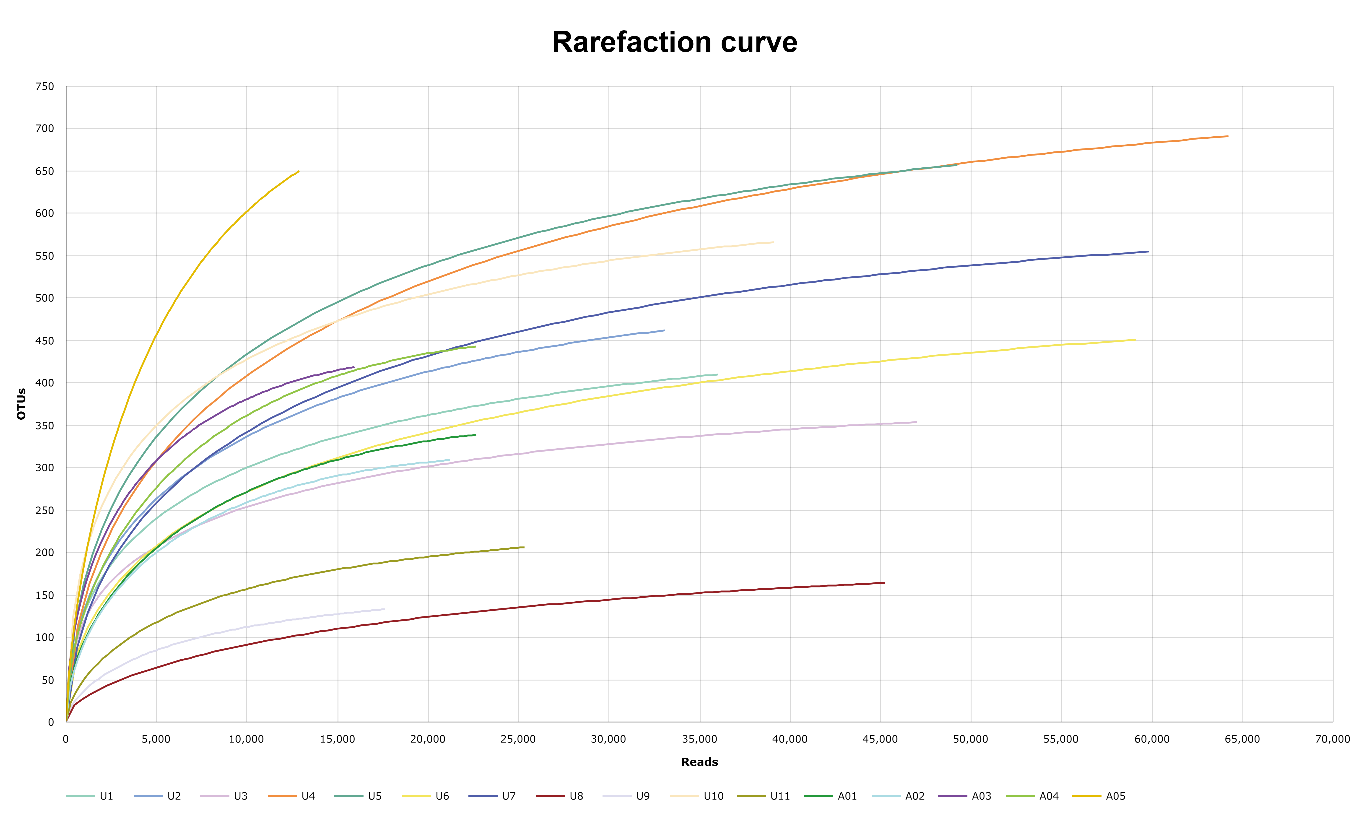


Figure S2. Illustrated the rarefaction curves derived from the read and OTU counts of 5 sampling sites on Mt. Brennkogel in the Eastern Alps (Austria) and 11 sampling sites on the Rwenzori Mountains ( Uganda).


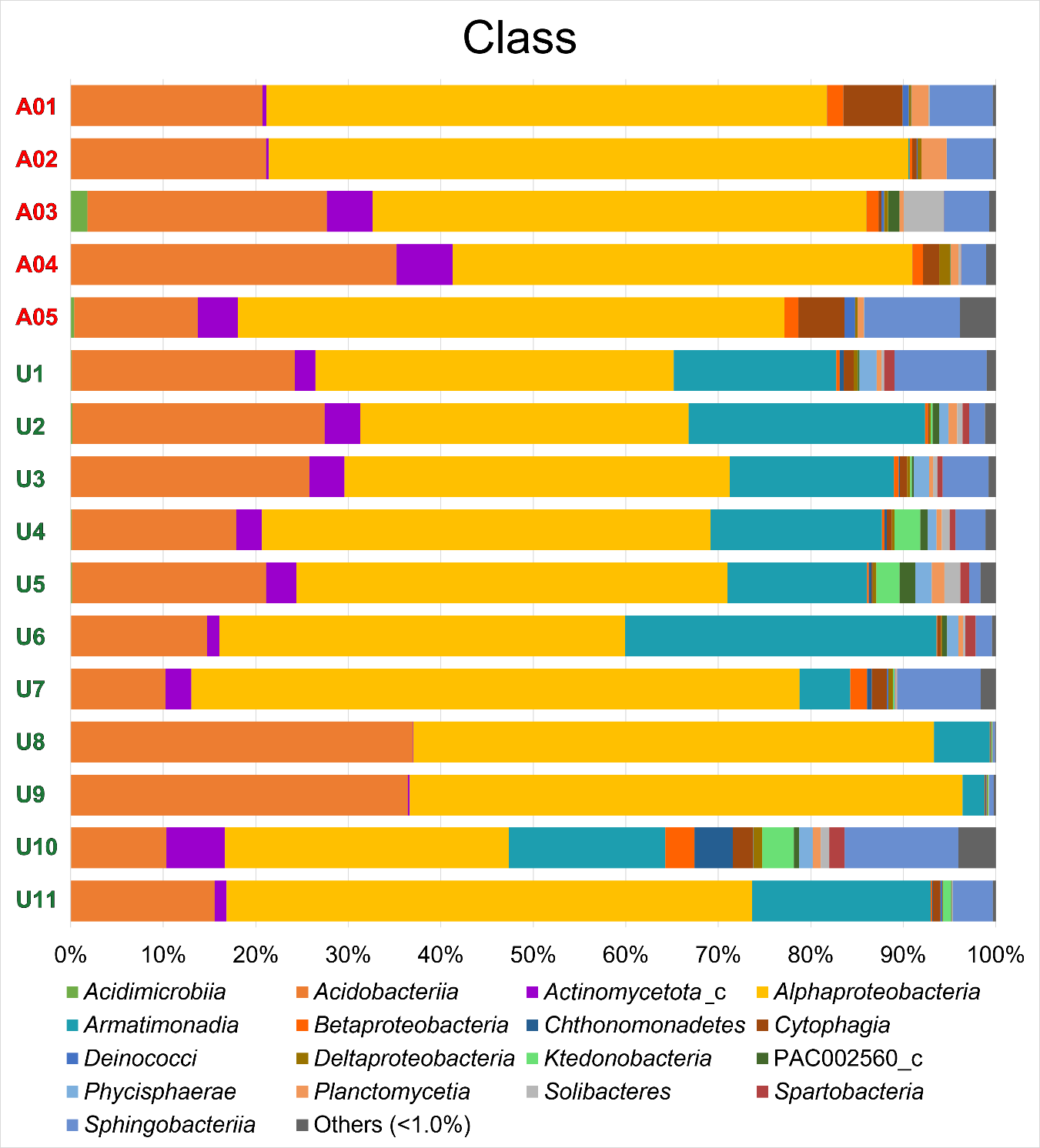


Figure S3. The distribution of bacterial classes among OTUs in lichen samples collected from the Eastern Alps (Austria, A01 to A05) and the Rwenzori Mountains (Uganda, U1 to U11) was examined.


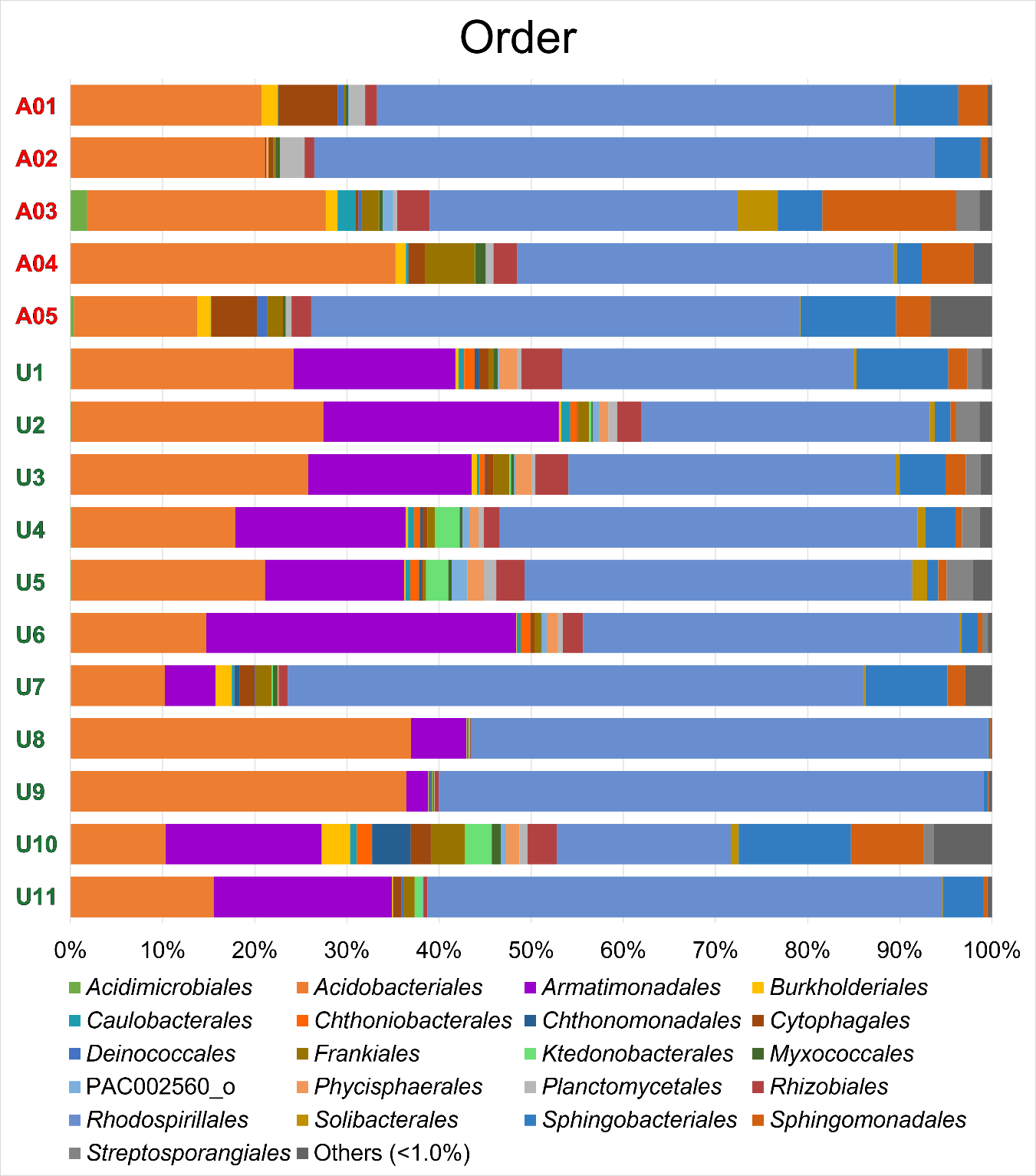


Figure S4. The distribution of bacterial orders among OTUs in lichen samples collected from the Eastern Alps (Austria, A01 to A05) and the Rwenzori Mountains (Uganda, U1 to U11) was examined.


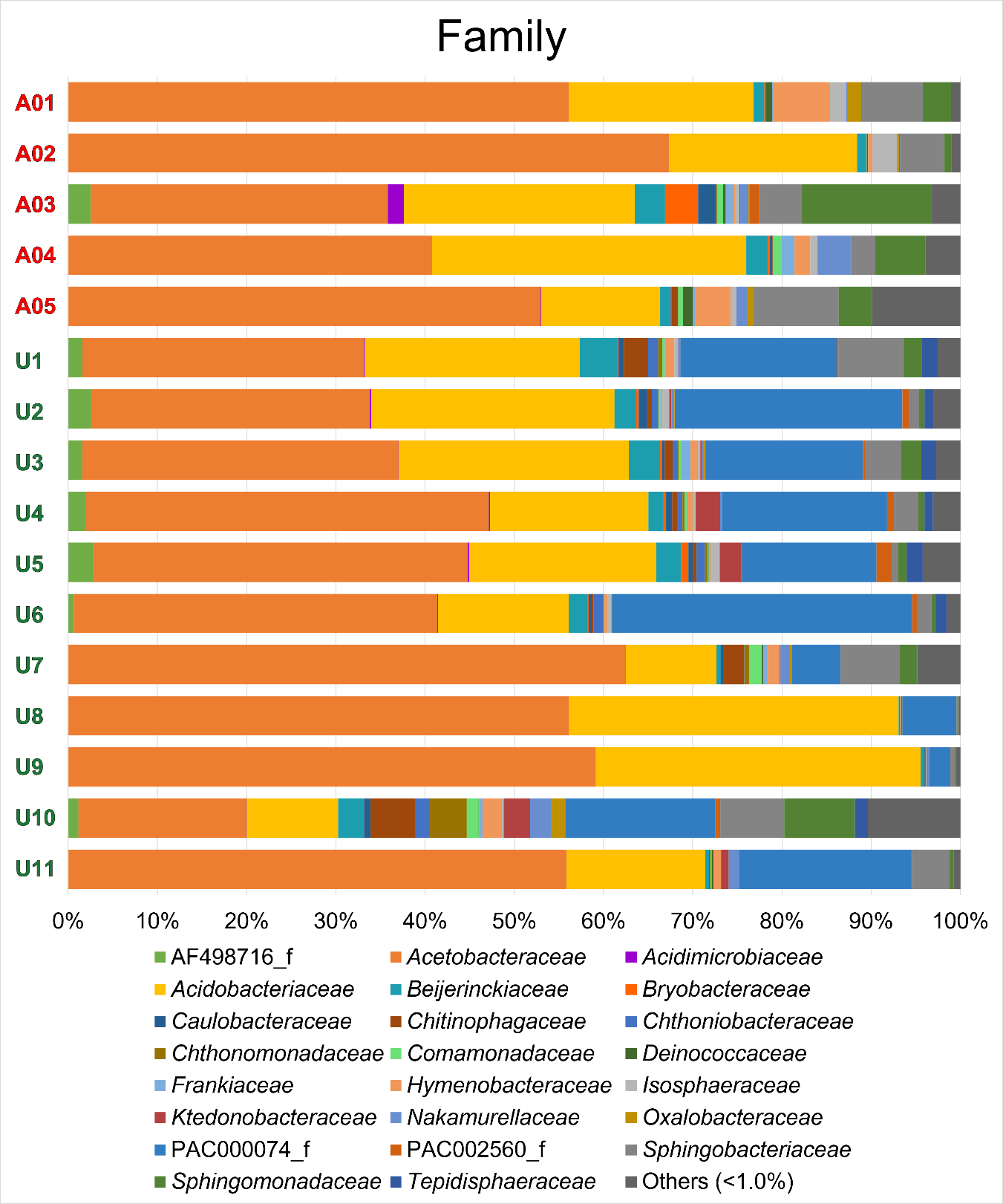


Figure S5. The distribution of bacterial families among OTUs in lichen samples collected from the Eastern Alps (Austria, A01 to A05) and the Rwenzori Mountains (Uganda, U1 to U11) was examined.


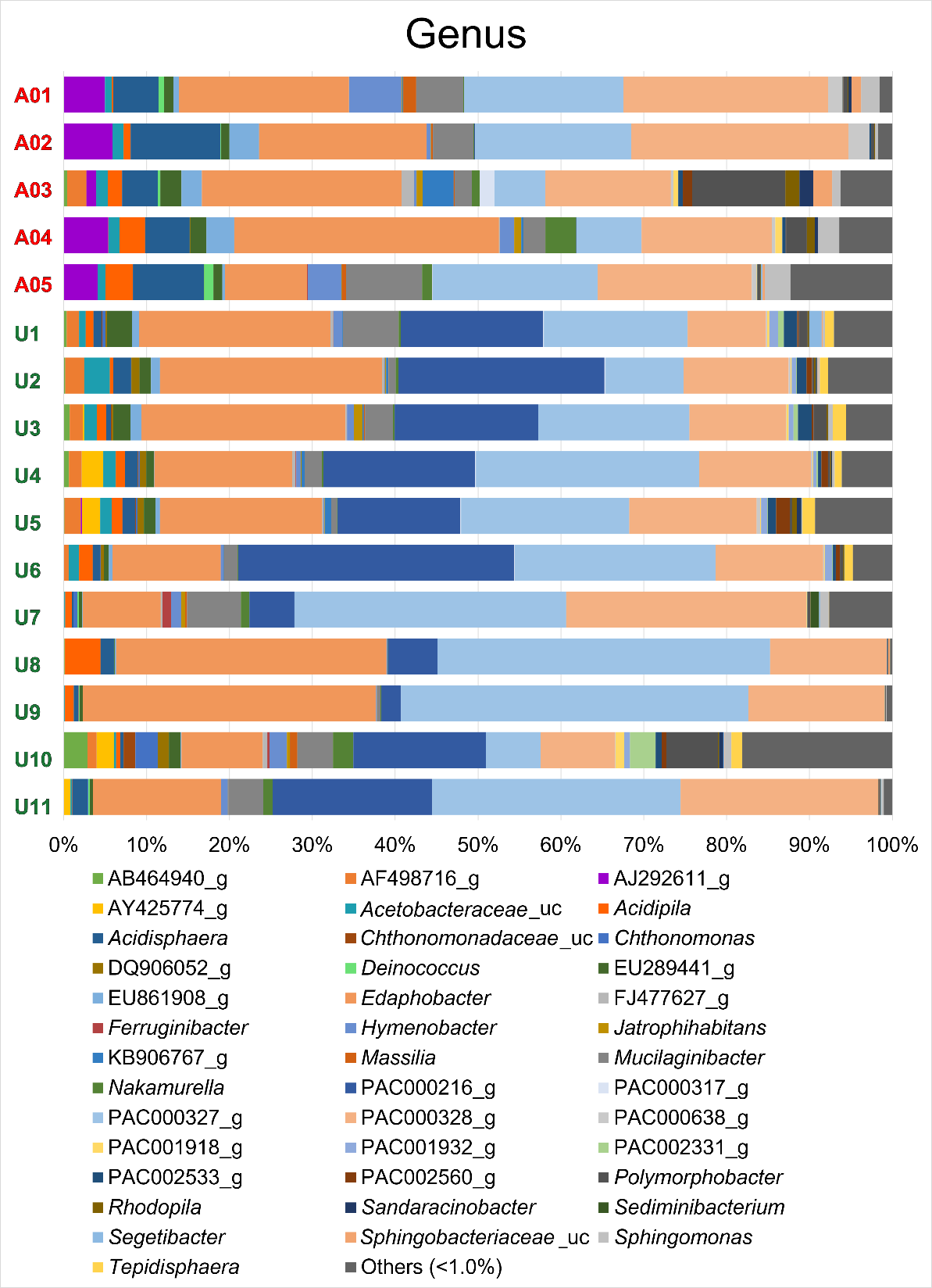


Figure S6. The distribution of bacterial genera among OTUs in lichen samples collected from the Eastern Alps (Austria, A01 to A05) and the Rwenzori Mountains (Uganda, U1 to U11) was examined.


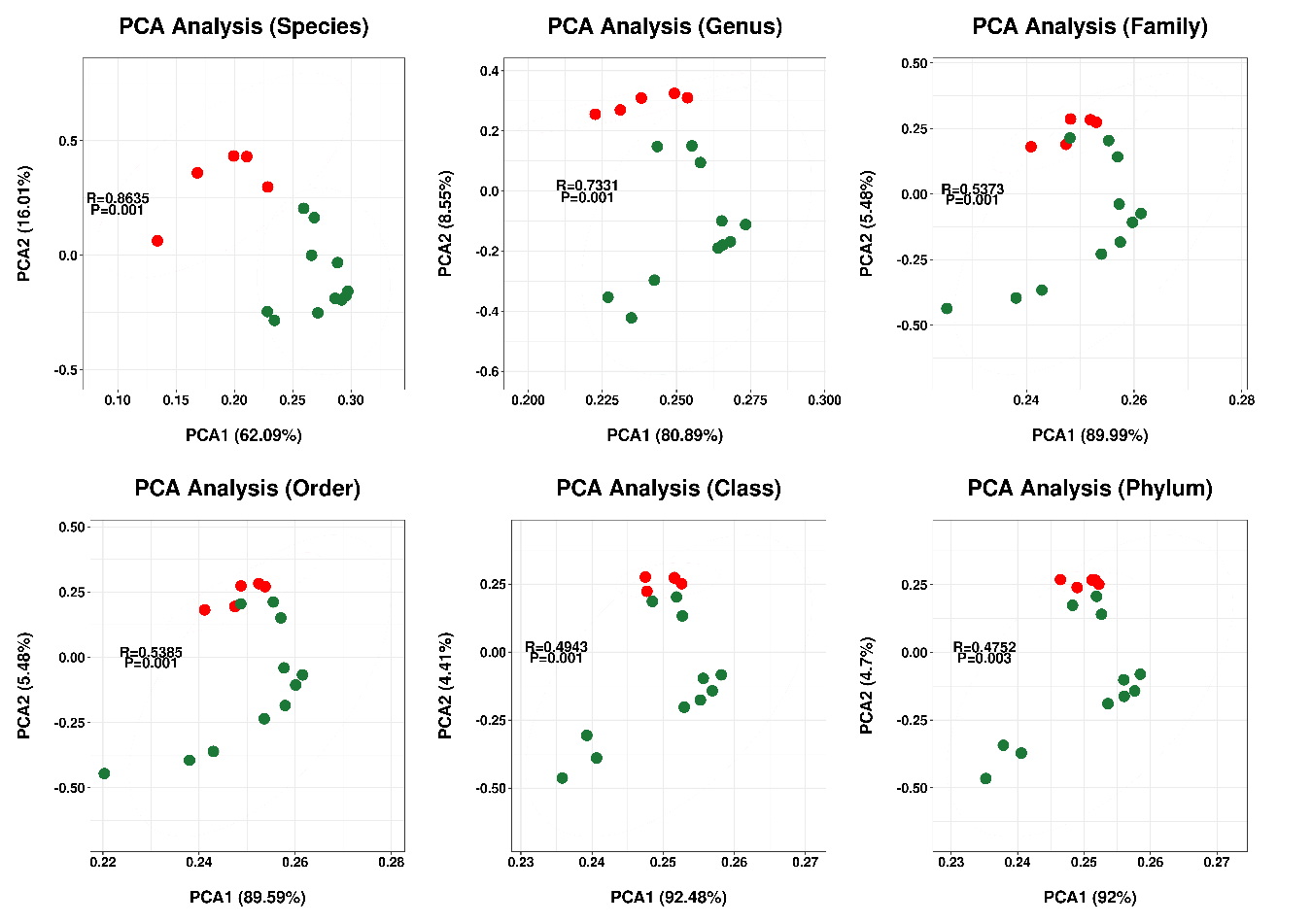


Figure S7. PCA plots were generated to visualize the distribution of OTU-derived species (top left), genera (top middle), families (top right), orders (bottom left), classes (bottom middle), and phyla (bottom right) among lichen samples collected from Mt. Stanley of the Eastern Alps (Austria, denoted in red) and the Rwenzori Mountains (Uganda, denoted in green).


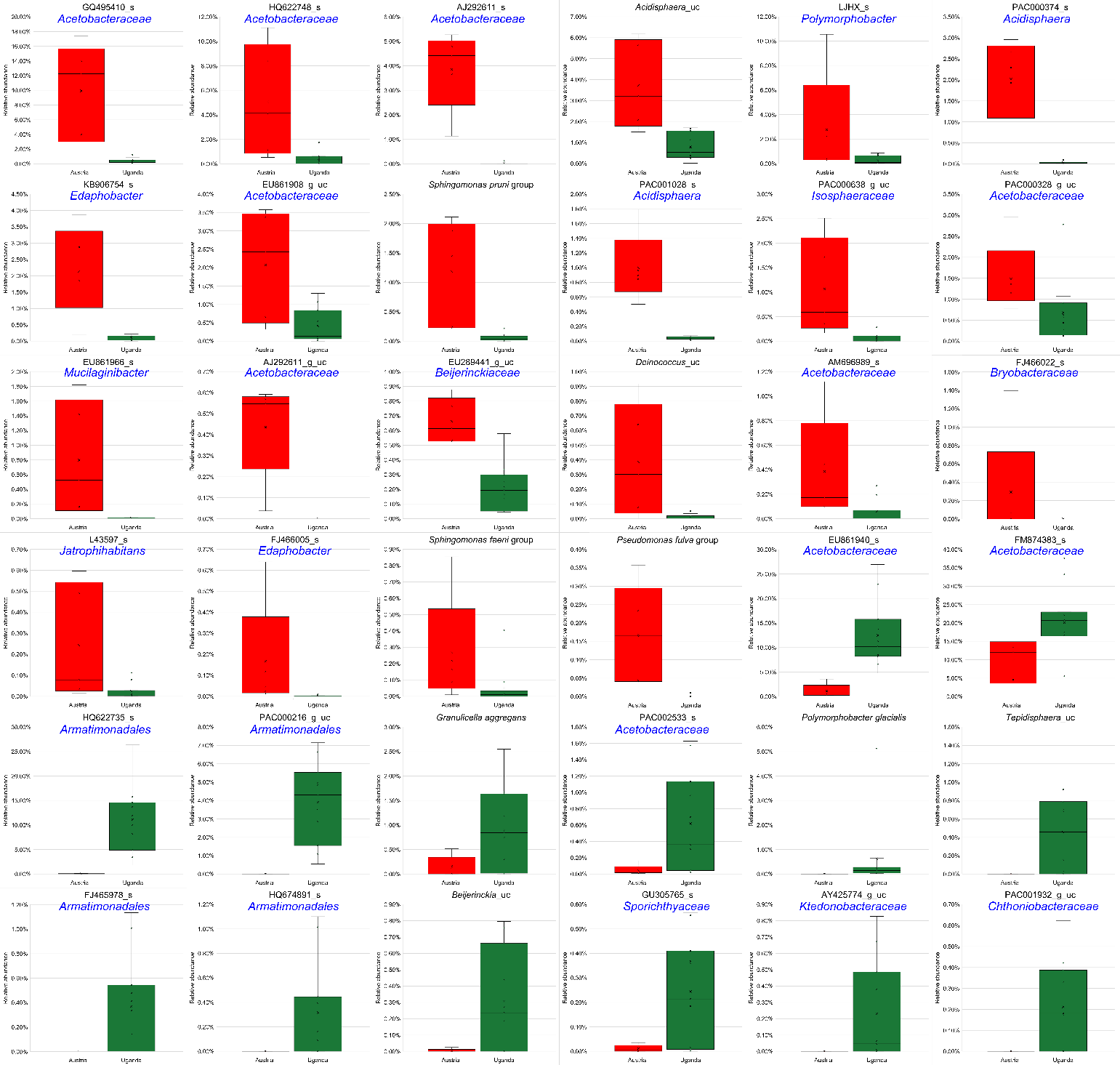


Figure S8. Statistically significant disparities (p < 0.05) in the relative abundances of indicator OTUs were assessed using ANCOM-BC between the Eastern Alps' Mt. Brennkogel (denoted in red) and the Rwenzori Mountains' Mt. Stanley (denoted in green) The names of potentially associated genera or phyla, if applicable, are displayed beneath the respective OTU identifiers.


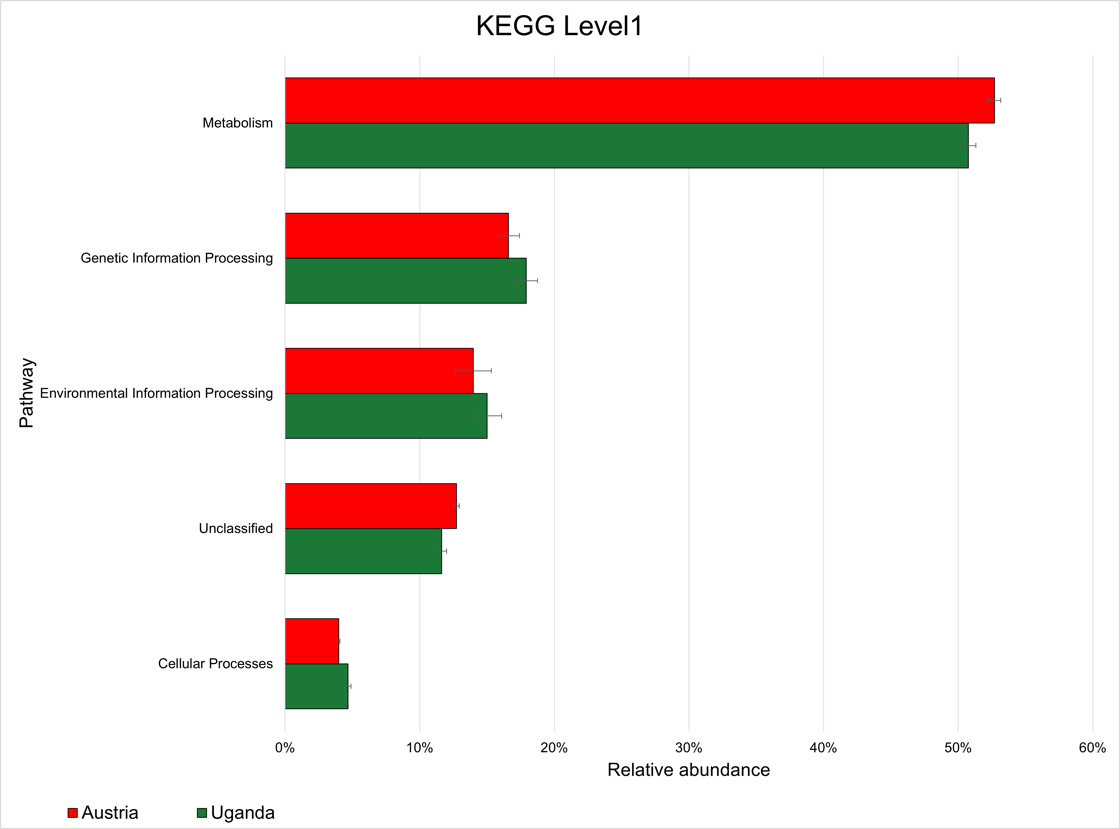


Figure S9. Metabolic pathways at KEGG Level 1 identified within the indicator OTUs originating from the Eastern Alps' Mt. Brennkogel (denoted in red) and the Rwenzori Mountains' Mt. Stanley (denoted in green).


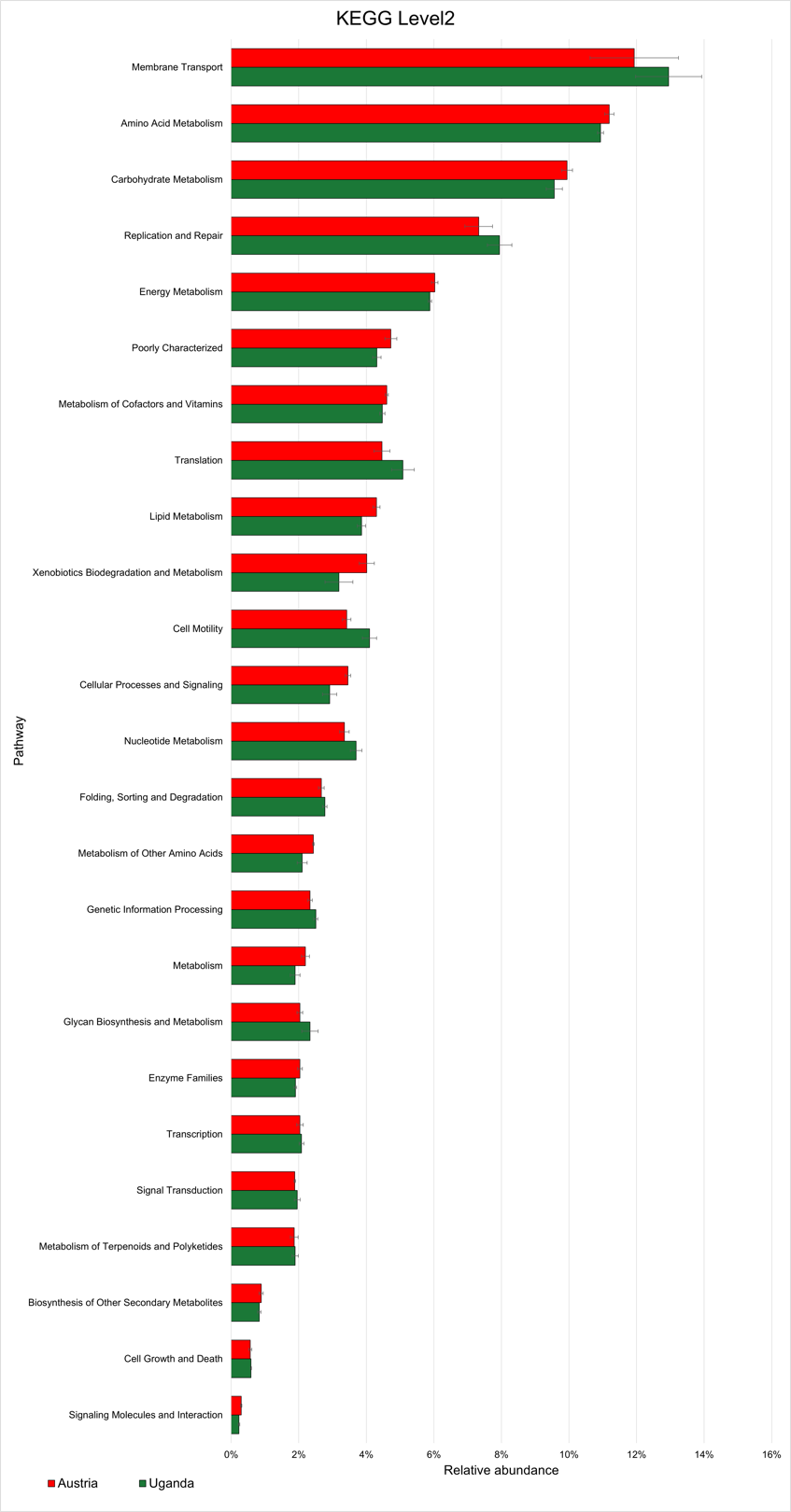


Figure S10. Metabolic pathways at KEGG Level 2 identified within the indicator OTUs originating from the Eastern Alps' Mt. Brennkogel (denoted in red) and the Rwenzori Mountains' Mt. Stanley (denoted in green).


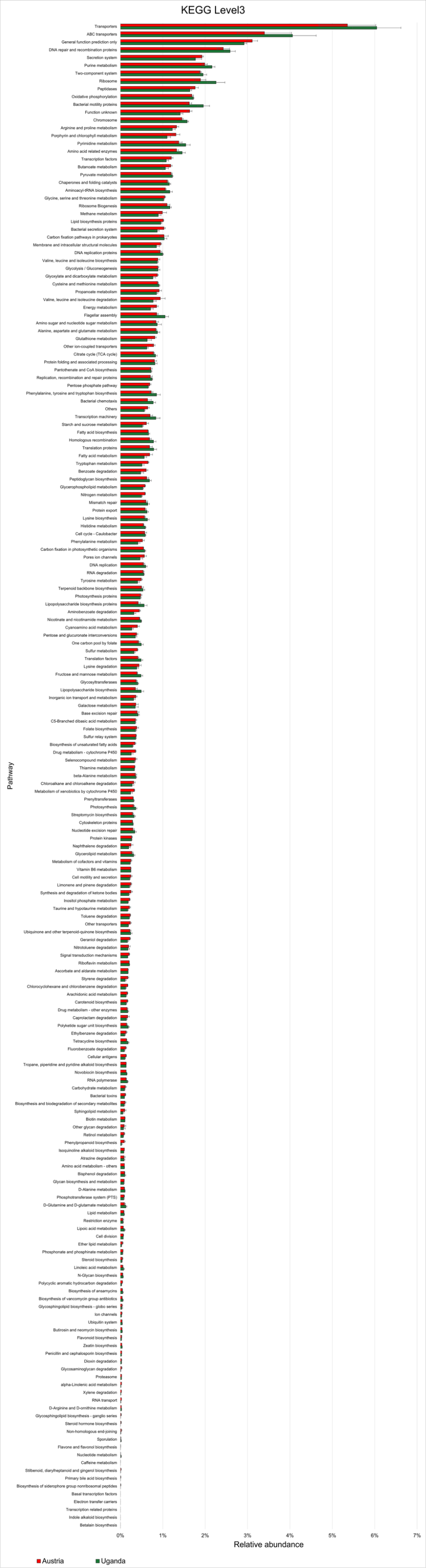


Figure S11. Metabolic pathways at KEGG Level 3 identified within the indicator OTUs originating from the Eastern Alps' Mt. Brennkogel (denoted in red) and the Rwenzori Mountains' Mt. Stanley (denoted in green).

(Figure S11**,** continued)


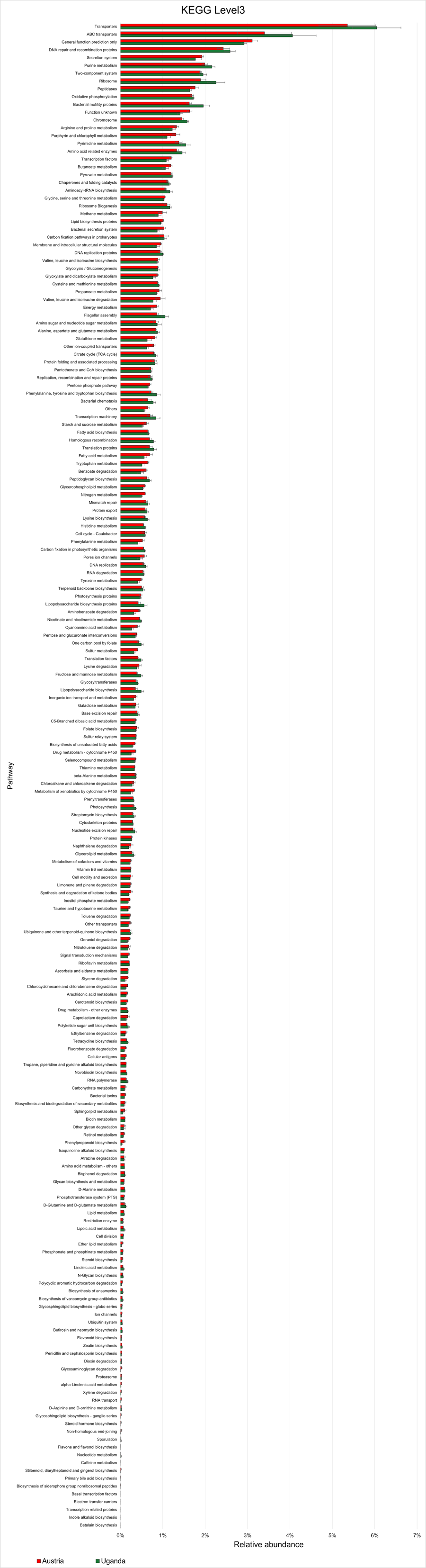

Supplement: Supplementary file 1 — Supplementary file1 (DOCX 6086 kb) Supplementary Materials: Supplementary materials can be accessed and downloaded from the provided link: https://static-content.springer.com/esm/xxx. Table S1: Provided are the BioProject numbers, DRA accession numbers, and BioSample accession numbers associated with the sequence datasets of the V3-V4 region that have been deposited in the public DDBJ database; Table S2: Provided are the accession numbers for sequences of near-full-length fungal 18S rRNA gene originating from the examined rock tripe lichen samples. The table also includes corresponding lengths, the most closely associated species with their respective accession numbers and lengths, and the corresponding similarity values (%); Table S3: Provided are the accession numbers for fungal ITS sequences which were deleted partial 18S and partial 28S ribosomal RNA sequences originating from the examined rock tripe lichen samples. The table also includes corresponding lengths, the most closely associated species with their respective accession numbers and lengths, and the corresponding similarity values (%); Table S4: Provided are the accession numbers for fungal ITS sequences with partial 18S and partial 28S ribosomal RNA sequences originating from the examined rock tripe lichen samples. The table also includes corresponding lengths, the most closely associated species with their respective accession numbers and lengths, and the corresponding similarity values (%); Table S5: Overall outlook of S2-S3-S4 only with top-hit species names; Table S6: Information of Umbilicaria aprina, U. rhizinata and U. africana in the database of NCBI; Table S7: Provided are the accession numbers for sequences of near-full-length algal 18S rRNA gene originating from the examined rock tripe lichen samples. The table also includes corresponding lengths, the most closely associated species with their respective accession numbers, and the corresponding similarity values (%).Figure S1: Photographs of [file 284_2024_3626_MOESM1_ESM.docx]
